# *CSSR*: assignment of secondary structure to coarse-grained RNA tertiary structures

**DOI:** 10.1107/S2059798322001292

**Published:** 2022-03-11

**Authors:** Chengxin Zhang, Anna Marie Pyle

**Affiliations:** a Department of Molecular, Cellular and Developmental Biology, Yale University, New Haven, CT 06511, USA; b Howard Hughes Medical Institute, Chevy Chase, MD 20815, USA; c Department of Computational Medicine and Bioinformatics, University of Michigan, Ann Arbor, MI 48109, USA; d Department of Chemistry, Yale University, New Haven, CT 06511, USA

**Keywords:** RNA, secondary-structure assignment, coarse-grained structure model, *CSSR* algorithm

## Abstract

RNA secondary-structure (rSS) assignment is one of the most routine forms of analysis of RNA 3D structures. However, traditional rSS assignment programs require full-atomic structures of the individual RNA nucleotides. This prevents their application to the modeling of RNA structures in which base atoms are missing. To address this issue, *Coarse-grained Secondary Structure of RNA* (*CSSR*), an algorithm for the assignment of rSS for structures in which nucleobase atomic positions are incomplete, has been developed. Using *CSSR*, an rSS assignment accuracy of ∼90% is achieved even for RNA structures in which only one backbone atom per nucleotide is known. Thus, *CSSR* will be useful for the analysis of experimentally determined and computationally predicted RNA 3D structures alike. The source code of *CSSR* is available at https://github.com/pylelab/CSSR.

## Abstract 2

*CSSR*, an algorithm for assigning secondary structures to RNA 3D structures with missing atoms, has been developed. The base-pair assignment accuracy is close to 90% for 3D structures in which only one atom per nucleotide can be empirically identified.

## 1. Introduction

In order to carry out their biological functions, many RNA molecules assemble into compact structures by forming networks of base-paired interactions, known as RNA secondary structure (rSS). Traditional rSS assignment programs such as *Dissecting the Spatial Structure of RNA* (*DSSR*; Lu *et al.*, 2015 ▸), *RNAview* (Yang *et al.*, 2003 ▸), *MC-Annotate* (Gendron *et al.*, 2001 ▸), *FR*3*D* (Sarver *et al.*, 2007 ▸) and *RNApdbee* (Zok *et al.*, 2018 ▸) require full-atomic structures in order to specifically identify individual nucleotides of modeled base pairs. Here, we refer to ‘rSS assignment’ as the determination of specific base pairings from the 3D coordinates of solved RNA structures or models. The accurate computational assignment of rSS is particularly important for monitoring and analyzing specific changes in secondary structure that occur during simulations of RNA 3D conformational change or folding pathways (Ding *et al.*, 2008 ▸). While there are empirical methods for determining rSS states from experimental data, such as *SHAPE-MaP* (Siegfried *et al.*, 2014 ▸) and *DMS-MaP* (Zubradt *et al.*, 2017 ▸), it remains important to develop orthogonal computational methods for assigning rSS from full-atomic structures.

One barrier to accurate rSS assignment is that many experimental and computational RNA 3D structures are relatively coarse-grained, *i.e.* there are regions of the structure that are not known with certainty, or there are regions (or atoms) that are completely missing. For example, among the experimentally determined RNA structures deposited in the PDB, approximately 5.6% of the RNA chains only contain P atoms. Meanwhile, while there are a few programs such as *FARFAR* (Watkins *et al.*, 2020 ▸) that sample full-atomic RNA structures, many popular RNA structure-prediction programs (Gherghe *et al.*, 2009 ▸; Tan *et al.*, 2006 ▸) mainly or solely represent predicted structures as coarse-grained models. For example, 3*dRNA* (Wang *et al.*, 2017 ▸) can represent each nucleotide by six atoms (P, C4′ and C1′ on the backbone and C2, C4 and C6 on nucleobases), *IsRNA* (Zhang & Chen, 2018 ▸) includes five atoms per pyrimidine nucleotide (P, C4′ and three nucleobase atoms) and four atoms per purine nucleotide (P, C4′ and two nucleobase atoms), *SimRNA* (Rother *et al.*, 2012 ▸) includes three types of atoms (P, C1′ and the glycosidic N of the nucleobase) and *NAST* (Jonikas *et al.*, 2009 ▸) only samples conformations by monitoring the position of the C3′ atoms. The resulting lack of full-atomic information complicates the follow-up structural analyses, including rSS assignments.

Previous efforts have been made to assign rSS to reduced representations of RNA structures. For example, the *ClaRNA* server (Waleń *et al.*, 2014 ▸) can reconstruct missing atoms before rSS assignment, as long as at least three base atoms are present for each nucleotide. It is, however, unable to handle coarse-grained structures containing two or fewer base atoms, which is a common case for low-resolution experimental structures and coarse-grained computational models. Perhaps the first program that can assign rSS for highly coarse-grained RNA structures is *pdb*2*ss*, which is a submodule of the *RNA-align* package (Gong *et al.*, 2019 ▸) that is used for tertiary-structure alignment. The *pdb*2*ss* program infers base pairs according to the distances between backbone atoms. Since it does not consider orientations between nucleotide pairs, its assignment accuracy is low, especially when only phosphate atoms are available, as shown in later sections of this paper.

To address these issues, we developed *CSSR*, which is an automated algorithm for rSS assignment that is applicable to any RNA PDB structure with one or any combination of the following ten atom types: the phosphate atom (P), the eight heavy atoms on the sugar ring (C5′, C4′, C3′, C2′, C1′, O5′, O4′ and O3′) and the glycosidic N atom of the nucleobase. The rSS assignment is achieved by computing the agreement of pseudo-bond lengths, pseudo-bond angles and dihedral angles formed by constituent atoms between an input structure and the standard length/angle/dihedral values from statistics of canonical base pairs in high-resolution RNA structures. The *CSSR* program can be used for the ultrafast calculation of base-pairing energy terms during RNA folding and refinement simulations (Wang *et al.*, 2017 ▸; Rother *et al.*, 2012 ▸, Jonikas *et al.*, 2009 ▸) and for generating training labels for low-resolution experimental structures for machine-learning-based rSS predictors (Singh *et al.*, 2019 ▸).

## 2. Materials and methods

### 2.1. *CSSR* score calculation

For a given input atomic RNA structure, *CSSR* first identifies nucleotide pairs that satisfy the following two criteria: firstly the nucleotide should have at least one of the ten atom types considered by *CSSR* and secondly the nucleotide type should be compatible with canonical base pairing, defined as Watson–Crick (A:U or C:G) and wobble (G:U) pairs. For each nucleotide pair *i* and *j* that satisfies these criteria, the *CSSR* score is calculated to indicate the base-pairing potential:

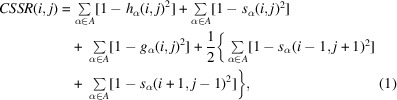

where 

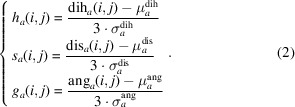




**Figure 1:**
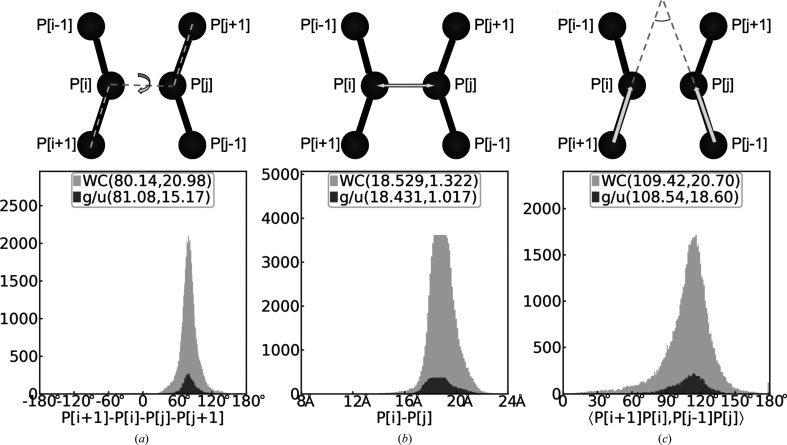
Illustration of the geometry terms [dihedral angle dih_P_(*i*, *j*) (*a*), distance dis_P_(*i*, *j*) (*b*) and angle ang_P_(*i*, *j*) (*c*)] included in *CSSR* score calculation for nucleotide pair *i* and *j* in an input RNA structure with only a P atom. Each ‘P’ in the upper panels represents the P atom of a single nucleotide; a solid black bar connecting two P atoms means the two nucleotides are adjacent nucleotides in the same strand. The lower panels are the background distribution of these geometry terms among experimental RNA structures. Distributions for Watson–Crick (WC) and G:U wobble (g/u) base pairs are shown in light and dark gray, respectively, while the mean and standard deviation of the distributions are listed within the parentheses in the legend. The distribution of geometry terms for other atom types are shown in Supplementary Fig. S1. Here, P[i], P[i+1] and P[i-1] refer to the P atoms of nucleotide *i* and those of the previous and subsequent nucleotide along the sequence.

Here, *A* = {P, C5′, C4′, C3′, C2′, C1′, O5′, O4′, O3′, N} is the set of atom types considered; dih*_a_* (*i*, *j*), dis*_a_* (*i*, *j*) and ang*_a_* (*i*, *j*) are inter-nucleotide dihedral angles, inter-nucleotide distances and inter-atomic angles, respectively, between nucleotides *i* and *j* for atom type *a* as illustrated in Fig. 1 ▸; 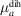 , 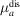 and 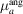 are their expected values, while 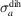 , 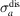 and 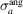 are the standard deviations for the dihedrals, distances and angles of their background distribution in experimental structures (Supplementary Fig. S1). If a certain dihedral/distance/angle cannot be calculated due to missing atoms, the respective term for the atom type is ignored for this nucleotide pair. In most RNA structures a base pair rarely exists as a singleton; instead, it is more commonly observed within helices, where the base pair can stack with a neighboring pair (or two neighboring base pairs) formed by adjacent nucleotides. Therefore, in *CSSR*(*i*, *j*), distances between *i* and *j*, between *i* + 1 and *j* − 1, and between *i* − 1 and *j* + 1 are all considered for each atom type. Meanwhile, the geometry definition of 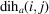 and 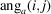 already considers the coordinates of nucleotides that are adjacent in the sequence. In (1), each geometry term has equal weight, because attempts to tune the weights among different terms did not result in more accurate rSS assignments.

### 2.2. Post-processing of *CSSR* scores

Since one nucleotide cannot simultaneously form Watson–Crick or wobble pairings with two or more nucleotides, it is necessary to filter *CSSR* scores to remove conflicting base pairs. To this end, all nucleotide pairs with *CSSR* scores ≥0.5 are listed in descending order of their scores. Here, the *CSSR* score cutoff of 0.5 is chosen as it provides a good balance between precision and recall for almost all atom types (black dots in Supplementary Fig. S2). Nucleotide pairs are then iteratively excluded from this list if one or both nucleotides overlap with any pairs that rank higher on the list. The remaining pairs in the list will be the final base pairs assigned by *CSSR*. This post-processing step does not use dynamic programming such as that implemented by the Zuker (Zuker & Stiegler, 1981 ▸) or Nussinov (Nussinov & Jacobson, 1980 ▸) algorithms, and is therefore capable of generating pseudo-knotted structures, as exemplified by Supplementary Fig. S3.

## 3. Results and discussion

### 3.1. Data set

*CSSR* is benchmarked on 361 nonredundant RNA chains collected from the PDB. This collection of RNAs was selected based on the following criteria. Firstly, each chain has 30–700 nucleotides and at least ten intra-chain canonical base pairs assigned by *DSSR* (Lu *et al.*, 2015 ▸). Secondly, only structures with resolution better than 4 Å are included so that *DSSR* can be used to accurately assign the ground-truth base pairs. Finally, similar to previous studies (Hanumanthappa *et al.*, 2020 ▸; Singh *et al.*, 2019 ▸), any two chains in the data set share <80% sequence identity, which is the minimal sequence-identity cutoff by *CD-HIT-EST* (Huang *et al.*, 2010 ▸).

### 3.2. Overall performance of *CSSR* on experimental 3D structures

**Figure 2:**
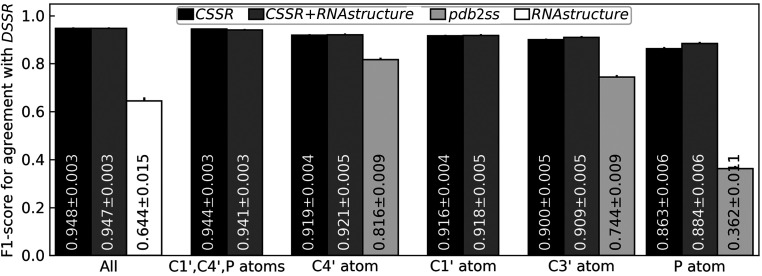
Average F1-score of rSS assignment by *CSSR* (black), *CSSR* + *RNAstructure* (dark gray), *pdb*2*ss* (light gray) and *RNAstructure* (white) for different atom types. ‘All’ means using all atoms for *CSSR* or sequence-based prediction without atomic coordinates for *RNAstructure*. The values within the bars are the average and standard error of mean (SEM) of per-target F1-scores. The error bars show the SEM values. F1-scores for other atom types are shown in Supplementary Tables S1 and S2.

As shown in Fig. 2 ▸, using C4′, C3′ or P atoms only, the rSS assigned by *CSSR* achieves an agreement of 0.919, 0.900 and 0.863, respectively, in terms of F1-score (see Section S1 for the definition) relative to the ground-truth assignment. These levels of agreements are 13%, 21% and 138% higher than those achieved by *pdb*2*ss*, which is the only existing rSS assignment program for coarse-grained RNA structures. Similar conclusions can be reached based on the Matthews correlation coefficient (MCC) instead of F1-score (Table 1 ▸). To put this into perspective, sequence-based rSS prediction by *RNAstructure* (Reuter & Mathews, 2010 ▸) using only thermodynamic parameters achieves an F1-score of 0.644 on this data set, indicating that accurate assignment of rSS for this data set is not trivial. In this comparison, among the programs included in the *RNAstructure* package for rSS prediction, the *ProbablePair* program is chosen due to its slightly higher F1-score compared with those from other programs, including *ProbKnot* (F1-score = 0.636), *Fold* (F1-score = 0.610) and *CycleFold* (F1-score = 0.408).

Notably, using only three atoms per nucleotide (P, C4′ and C1′), *CSSR* achieves a high agreement (F1-score = 0.944) with ground-truth assignment, which is derived by *DSSR* (Lu *et al.*, 2015 ▸) using the full-atomic RNA structures. This F1-score is almost the same as that achieved by *CSSR* using a full-atomic structure (F1-score = 0.948) and is comparable to the agreements among full-atomic rSS assignment programs (F1-score = 0.965 for *DSSR* versus *RNAView*; F1-score = 0.942 for *DSSR* versus *MC-Annotate*; Table 1 ▸). These data suggest that three backbone atoms are sufficient to accurately define the local geometry of an RNA structure.

It is more challenging to use the P atom than any other atom for rSS assignment by either *CSSR* or *pdb*2*ss*. This is because the interatomic distance in a canonical base pair is farthest for the P atom compared with all other atom types (Supplementary Fig. S1). Consequently, the distances, dihedrals and angles calculated using P atoms have the largest variations (Supplementary Fig. S1), which makes rSS assignment challenging. We tested whether rSS assignment for the P atom can be improved by combining *CSSR* and *RNA­structure* through weighted averaging of their assignment/prediction scores, as these two programs are based on completely different principles. As shown in Table 1 ▸, this strategy only leads to a minor improvement of 2% in F1-score under optimal weights of 0.8 and 0.2 for *CSSR* and *RNAstructure*, respectively, while the F1-score for other atom types show little to no improvement. Moreover, the inclusion of *RNAstructure* significantly slows down *CSSR*: for example, *CSSR* itself only needs 0.05 s for *Lactococcus* group II intron (PDB entry 5g2x chain *A*; 692 nucleotides) but needs 18 s to include *RNAstructure*. Therefore, in this work, we use *CSSR* without *RNAstructure* as the default rSS assignment, although *CSSR* + *RNAstructure* is offered as an optional feature in the *CSSR* standalone program.

While *CSSR* assigns both Watson–Crick base pairs (A:U and G:C) and wobble base pairs (G:U), the accuracies of Watson–Crick pair assignments are consistently higher than those for wobble pairs for all atomic types (Supplementary Table S3). This is probably due to the much smaller number of wobble base pairs available in experimental structures that can be used to train *CSSR* (Supplementary Fig. S1). Similarly, due to limited training structures, the current *CSSR* method cannot assign Hoogsteen/sugar edge base pairs, which are even rarer than wobble base pairs. As more and more experimental RNA structures are determined, it is likely that a future version of *CSSR* retrained on more structures could improve the assignment accuracies for these non-Watson–Crick base pairs.

### 3.3. Performance of *CSSR* on predicted RNA structure models

**Figure 3:**
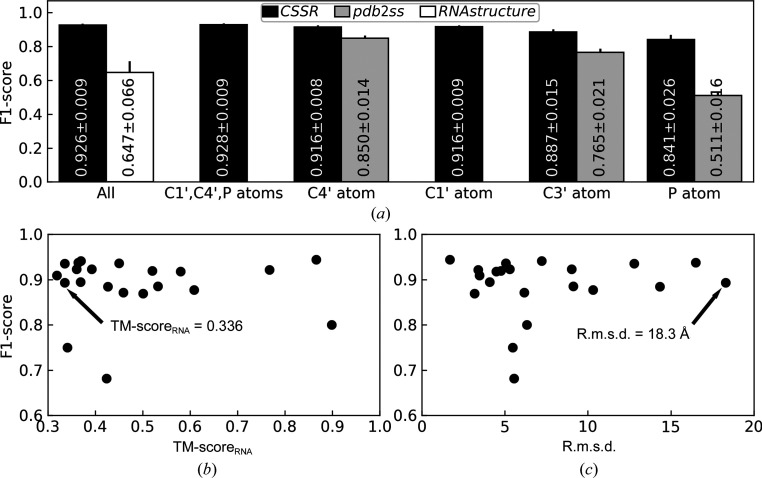
(*a*) Average F1-score of rSS assignment for predicted 3D structures. The error bars show the SEM values. The ground-truth rSS assignment was obtained by running *DSSR* for the full-atomic native structures. The F1-scores for other atom types are shown in Supplementary Tables S4 and S5. (*b*, *c*) The rSS assignment F1-score versus the quality of 3D structure models in terms of TM-score_RNA_ (*b*) or r.m.s.d. (*c*), where the glycine riboswitch is indicated by an arrow.

We further examined the ability of *CSSR* to assign rSS to computationally predicted structures, which is one of the important motivations for developing *CSSR*. To this end, we collected all 21 modeling targets from a recent community-wide RNA puzzle challenge (Magnus *et al.*, 2020 ▸), which is publicly available from https://github.com/mmagnus/RNA-Puzzles-Standardized-Submissions. This data set includes 15 monomeric RNAs, five RNA dimers and one RNA octamer. The modeling targets range from 41 to 188 nucleotides. Each target has up to 107 predicted structure models, among which the structure model with the best TM-score_RNA_ is selected for rSS assignment analysis. Here, TM-score_RNA_ is a sequence-length-independent metric previously developed to quantify the overall similarity between two RNA 3D structures (Gong *et al.*, 2019 ▸). TM-score_RNA_ ranges between 0 and 1, with higher TM-score_RNA_ corresponding to higher similarity. As shown in Fig. 3 ▸(*a*), even when using predicted 3D structure models as input, *CSSR* still achieves very high rSS assignment agreement with the native rSS (average F1-score = 0.926 for full-atomic models and F1-score = 0.916, 0.916 or 0.887 using C4′, C3′ or P atoms only). This level of agreement between native rSS and the rSS assignment for predicted structure models is similar to that achieved by existing full-atomic rSS assignment programs (average F1-score = 0.934, 0.931, 0.925 and 0.901 for *DSSR*, *ClaRNA*, *RNAView* and *MC-Annotate*, respectively; Supplementary Table S4). This suggests the usefulness of *CSSR* even for low-resolution 3D structure models.

**Figure 4:**
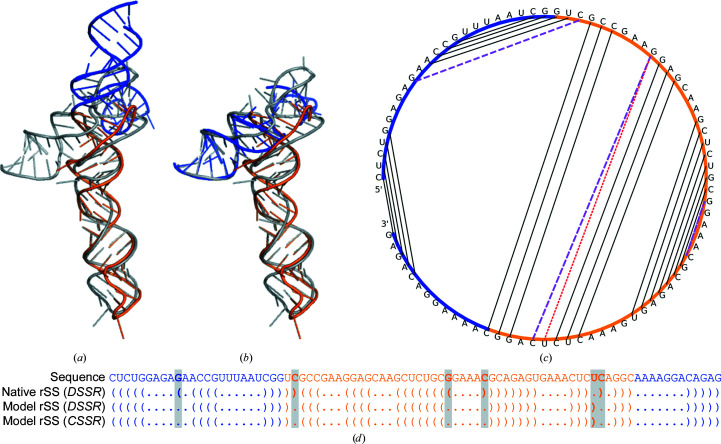
3D structure and rSS of a glycine riboswitch. (*a*) The RNA puzzle structure model (the first 24 and last 12 nucleotides are in blue; the middle 48 nucleotides are in orange) superimposed on the experimental structure (gray) as a whole chain. (*b*) The blue and orange parts of the structure model separately superimposed on the experimental structure with r.m.s.ds of 10.6 and 3.8 Å, respectively. (*c*) Schematic of rSS. Base pairs that are in the experimental 3D structure but not in the 3D structure model are shown by magenta dashed lines. The base pair that is in the structure model but not in the experimental 3D structure is shown by a red dotted line. Base pairs common to experimental and computational 3D structures are shown by black solid lines. (*d*) Sequence, rSS of the experimental structure (from *DSSR*) and rSS of the structure model, where assignments by *DSSR* and by *CSSR* are identical. The colors of the sequences correspond to the colors of the corresponding structure models in (*a*) and (*b*). Nucleotides with different base pairing in the experimental and computational 3D structures are shaded.

Perhaps surprisingly, the rSS assignment accuracy has little correlation with the correctness of the global topology (TM-score_RNA_ and r.m.s.d.) of the input 3D structure model, with Pearson correlation coefficients (PCCs) of −0.016 and 0.111, respectively (Figs. 3 ▸ *b* and 3 ▸ *c*). This is largely because RNA models with low global 3D structure quality can still have a high degree of rSS agreement with the native structure. As a case study, we examined the glycine riboswitch from RNA puzzle problem 3. The structure model has a TM-score_RNA_ of 0.336 and an r.m.s.d. of 18.3 Å relative to the experimental structure (PDB entry 3owi chain *A*; Fig. 4 ▸ *a*). The main reason for the dissimilarity between the experimental and computationally determined structures is that the placement of the first 24 and last 12 nucleotides (blue in Figs. 4 ▸ *a* and 4 ▸ *b*) was incorrect in the computational model, although the remaining 48 nucleotides adopted the correct topology (orange in Figs. 4 ▸ *a* and 4 ▸ *b*). Despite an inaccurate 3D structure model, the rSS was largely modeled correctly (Figs. 4 ▸ *c* and 4 ▸ *d*), with only three missing base pairs and one incorrectly included base pair in the 3D model. Since the top RNA puzzle algorithms (Biesiada *et al.*, 2016 ▸; Watkins *et al.*, 2020 ▸; Wang *et al.*, 2017 ▸; Xu *et al.*, 2014 ▸) introduce strong rSS restraints during the conformation-sampling simulation, the resulting RNA 3D structure models, including that analyzed in Fig. 4 ▸, usually preserve a high degree of rSS consistency with the native structure. Nonetheless, our case study exemplifies the difficulty of modeling non-base-paired interactions to derive a correct 3D model from the rSS.

### 3.4. Performance of *CSSR* on low-resolution experimental RNA structures

We further tested *CSSR* on 16 low-resolution RNA experimental structures for which high-resolution full-atomic structures of the same RNAs are also available. All low-resolution structures contained only P atoms. On average, *CSSR* achieves an F1-score of 0.884 to the ground-truth rSS assigned by *DSSR* to the high-resolution structure (Supplementary Table S6). This is much higher than that achieved by *pdb*2*ss* (F1-score = 0.495) and sequence-based rSS prediction by *RNAstructure* (F1-score = 0.697). These data confirm the applicability of *CSSR* to low-resolution experimental data.

## 4. Conclusion

We developed *CSSR*, a new rSS assignment algorithm for detecting base pairs in RNA 3D structures. To our knowledge, *CSSR* is the one of only two algorithms available for rSS assignment in RNA 3D structures with missing atoms, and the only algorithm with 90% rSS assignment accuracy. The high accuracy of *CSSR* and its robustness, regardless of the input structure quality, makes *CSSR* a useful tool for modeling the base pairing within both experimental and computationally determined RNA structures. Moreover, the base-pairing score of *CSSR* (1) is easy to calculate and differentiable, making it easy to incorporate into RNA 3D structure-simulation programs (Wang *et al.*, 2017 ▸; Rother *et al.*, 2012 ▸; Jonikas *et al.*, 2009 ▸) as an energy term. The current version of *CSSR* focuses on the assignment of canonical base pairs. A natural extension would be the assignment of non-canonical base pairs. Work along this line is in progress.

## Supplementary Material

Evaluation metrics for rSS assignment, Supplementary Tables and Supplementary Figures. DOI: 10.1107/S2059798322001292/cb5131sup1.pdf

## Figures and Tables

**Table 1:** Average F1-score (average MCC) obtained by *CSSR*, *pdb*2*ss*, *RNAstructure*, *CSSR* + *RNAstructure*, *RNAView* and *MC-Annotate* for 361 benchmark RNAs Different columns represent different atom types. ‘All’ means using all atoms for *CSSR* and *RNAView* and using only sequence without atomic coordinates for *RNAstructure*. The value for *pdb*2*ss* is NA (not applicable) in this column because it can only perform single atom-based rSS assignment.

Method	All	C1′, C4′, P atoms	C4′ atom	C1′ atom	C3′ atom	P atom
*CSSR*	0.948 (0.949)	0.944 (0.945)	0.919 (0.920)	0.916 (0.917)	0.900 (0.901)	0.863 (0.864)
*pdb*2*ss*	NA	NA	0.816 (0.822)	NA	0.744 (0.758)	0.362 (0.412)
*RNAstructure*	0.644 (0.648)	NA	NA	NA	NA	NA
*CSSR* + *RNAstructure*	0.947 (0.948)	0.941 (0.942)	0.921 (0.922)	0.917 (0.919)	0.910 (0.911)	0.884 (0.886)
*RNAView*	0.965 (0.966)	NA	NA	NA	NA	NA
*MC-Annotate*	0.942 (0.944)	NA	NA	NA	NA	NA
