# Supplementary material for: CSSR: assignment of secondary structure to coarse-grained RNA tertiary structures
Source: Acta Crystallogr D Struct Biol. 2022 Mar 11;78(Pt 4):466–71. doi: 10.1107/S2059798322001292 (PMC8972804; doi:10.1107/S2059798322001292)
Supplement: Supplementary file 1 [file d-78-00466-sup1.pdf]

Acta Cryst D
STRUCTURAL BIOLOGY

**Volume 78 (2022)**

**Supporting information for article:**

# *CSSR*: assignment of secondary structure to coarse-grained RNA tertiary structures

**Chengxin Zhang and Anna Marie Pyle**

## S1. Evaluation metrics for rSS assignment

The accuracy of rSS assignment by CSSR can be measured by F1-score and Mathews Correlation Coefficient (MCC) for each target:

$$F1 = \frac{2 \cdot TP}{2 \cdot TP + FP + FN} \quad \ldots \text{(S1)}$$

$$MCC = \frac{TP \cdot TN - FP \cdot FN}{\sqrt{(TP+FP)(TP+FN)(TN+FP)(TN+FN)}} \quad \ldots \text{(S2)}$$

In the above equations, *TP* is the number of True Positive base pairs correctly assigned by CSSR; *FP* is the number of False Positive base pairs assigned by CSSR and not in the ground-truth DSSR assignment; *FN* is the number of False Negative base pairs in the ground-truth assignment that are missed by CSSR; *TN* is the number of True Negative nucleotide pairs that are neither assigned by CSSR nor by DSSR. Higher F1-score and higher MCC both correspond to better accuracy; both metrics equal to 1 for perfect assignment.

**Table S1** Target-specific F1-score of all atom types supported by CSSR for 361 benchmark RNAs.

# The RNA name consists of a 4-character PDB ID, followed by the chain ID. For example, 5g2xA means PDB ID 5g2x Chain A.

† "All" means using all supported atoms. CSSR supported any combination of its 10 types of atoms. Due to limitation of space, we only show two combinations: all atoms, and C1'C4'P atoms.

‡ "N" is atom N9 and N1 for purine and pyrimidine, respectively.

| RNA # | All † | C1'C4'P | P | O5' | C5' | C4' | C3' | C2' | C1' | O4' | O3' | N ‡ |
|---|---|---|---|---|---|---|---|---|---|---|---|---|
| 5g2xA | 0.661 | 0.667 | 0.527 | 0.577 | 0.624 | 0.593 | 0.574 | 0.575 | 0.577 | 0.562 | 0.550 | 0.389 |
| 6c4hA | 0.926 | 0.908 | 0.744 | 0.756 | 0.865 | 0.857 | 0.868 | 0.858 | 0.900 | 0.874 | 0.831 | 0.907 |
| 6hiwCA | 0.902 | 0.881 | 0.630 | 0.617 | 0.671 | 0.759 | 0.648 | 0.676 | 0.855 | 0.800 | 0.600 | 0.832 |
| 6chrA | 0.941 | 0.934 | 0.773 | 0.819 | 0.840 | 0.890 | 0.873 | 0.888 | 0.884 | 0.910 | 0.874 | 0.720 |
| 6hixAA | 0.901 | 0.906 | 0.602 | 0.662 | 0.762 | 0.783 | 0.784 | 0.735 | 0.846 | 0.783 | 0.734 | 0.855 |
| 6n7rR | 0.943 | 0.937 | 0.853 | 0.893 | 0.922 | 0.936 | 0.904 | 0.898 | 0.918 | 0.922 | 0.913 | 0.919 |
| 1fg0A | 0.947 | 0.939 | 0.866 | 0.887 | 0.886 | 0.905 | 0.907 | 0.912 | 0.913 | 0.914 | 0.892 | 0.875 |
| 5wlcL0 | 0.965 | 0.960 | 0.891 | 0.935 | 0.961 | 0.953 | 0.960 | 0.953 | 0.933 | 0.946 | 0.961 | 0.778 |
| 5zwnP | 0.911 | 0.905 | 0.868 | 0.862 | 0.895 | 0.894 | 0.877 | 0.880 | 0.886 | 0.894 | 0.850 | 0.845 |
| 6swe2 | 0.940 | 0.936 | 0.807 | 0.870 | 0.899 | 0.892 | 0.863 | 0.853 | 0.883 | 0.875 | 0.857 | 0.856 |
| 5j01A | 0.958 | 0.954 | 0.891 | 0.916 | 0.923 | 0.931 | 0.908 | 0.894 | 0.939 | 0.930 | 0.895 | 0.921 |
| 6agbA | 0.914 | 0.902 | 0.767 | 0.818 | 0.898 | 0.880 | 0.856 | 0.882 | 0.885 | 0.892 | 0.853 | 0.865 |
| 6q9a4 | 0.738 | 0.789 | 0.489 | 0.545 | 0.693 | 0.764 | 0.625 | 0.629 | 0.825 | 0.811 | 0.549 | 0.776 |
| 3bwpA | 0.954 | 0.935 | 0.877 | 0.905 | 0.931 | 0.898 | 0.902 | 0.880 | 0.915 | 0.903 | 0.877 | 0.918 |
| 3q1qB | 0.986 | 0.991 | 0.819 | 0.911 | 0.954 | 0.941 | 0.927 | 0.944 | 0.963 | 0.973 | 0.946 | 0.982 |
| 6ahuA | 0.924 | 0.911 | 0.838 | 0.821 | 0.899 | 0.900 | 0.896 | 0.862 | 0.895 | 0.885 | 0.904 | 0.790 |
| 6w2s0 | 0.729 | 0.717 | 0.571 | 0.566 | 0.602 | 0.604 | 0.645 | 0.556 | 0.786 | 0.635 | 0.583 | 0.650 |
| 6q954 | 0.775 | 0.800 | 0.600 | 0.640 | 0.676 | 0.791 | 0.647 | 0.645 | 0.756 | 0.758 | 0.705 | 0.867 |
| 5u4ja | 0.958 | 0.964 | 0.875 | 0.894 | 0.947 | 0.946 | 0.892 | 0.893 | 0.925 | 0.946 | 0.885 | 0.918 |
| 2a64A | 0.952 | 0.979 | 0.856 | 0.898 | 0.969 | 0.958 | 0.925 | 0.915 | 0.941 | 0.963 | 0.942 | 0.946 |
| 6g901 | 0.895 | 0.906 | 0.889 | 0.851 | 0.871 | 0.877 | 0.871 | 0.884 | 0.872 | 0.883 | 0.890 | 0.846 |
| 6sxoL5 | 0.929 | 0.915 | 0.667 | 0.725 | 0.889 | 0.874 | 0.842 | 0.876 | 0.915 | 0.903 | 0.837 | 0.919 |
| 5flxz | 0.962 | 0.948 | 0.865 | 0.850 | 0.930 | 0.929 | 0.903 | 0.899 | 0.941 | 0.935 | 0.930 | 0.967 |
| 6frk1 | 0.949 | 0.928 | 0.920 | 0.907 | 0.923 | 0.909 | 0.909 | 0.819 | 0.855 | 0.884 | 0.850 | 0.820 |
| 5a8lA | 0.849 | 0.857 | 0.571 | 0.611 | 0.693 | 0.831 | 0.687 | 0.733 | 0.677 | 0.839 | 0.758 | 0.741 |
| 1x8wB | 0.964 | 0.957 | 0.847 | 0.898 | 0.906 | 0.920 | 0.906 | 0.897 | 0.918 | 0.934 | 0.884 | 0.892 |
| 5tc1R | 0.711 | 0.697 | 0.602 | 0.667 | 0.619 | 0.677 | 0.687 | 0.661 | 0.650 | 0.672 | 0.702 | 0.456 |
| 5oql2 | 0.894 | 0.881 | 0.718 | 0.701 | 0.835 | 0.825 | 0.829 | 0.833 | 0.790 | 0.840 | 0.871 | 0.861 |
| 1y0qA | 0.970 | 0.970 | 0.904 | 0.934 | 0.934 | 0.963 | 0.933 | 0.933 | 0.939 | 0.955 | 0.963 | 0.833 |
| 6j6qL | 0.949 | 0.938 | 0.863 | 0.884 | 0.839 | 0.949 | 0.889 | 0.887 | 0.938 | 0.949 | 0.825 | 0.899 |
| 6p5n1 | 0.819 | 0.835 | 0.731 | 0.750 | 0.735 | 0.808 | 0.720 | 0.729 | 0.833 | 0.804 | 0.707 | 0.776 |
| 5zwmH | 0.883 | 0.902 | 0.831 | 0.850 | 0.843 | 0.876 | 0.840 | 0.850 | 0.855 | 0.885 | 0.765 | 0.793 |
| 5jupEC | 0.904 | 0.849 | 0.627 | 0.699 | 0.720 | 0.825 | 0.735 | 0.778 | 0.804 | 0.812 | 0.667 | 0.791 |
| 1u6bB | 0.982 | 0.972 | 0.917 | 0.924 | 0.953 | 0.933 | 0.942 | 0.943 | 0.942 | 0.942 | 0.935 | 0.942 |
| 6d904 | 0.737 | 0.755 | 0.633 | 0.597 | 0.643 | 0.691 | 0.691 | 0.453 | 0.667 | 0.692 | 0.586 | 0.692 |
| 4gmaZ | 0.906 | 0.887 | 0.756 | 0.810 | 0.835 | 0.836 | 0.821 | 0.850 | 0.883 | 0.850 | 0.857 | 0.779 |
| 6gyvA | 0.992 | 0.983 | 0.911 | 0.933 | 0.975 | 0.967 | 0.942 | 0.942 | 0.959 | 0.951 | 0.959 | 0.966 |

| | | | | | | | | | | | | |
|---|---|---|---|---|---|---|---|---|---|---|---|---|
| 5v3iA | 0.992 | 1.000 | 0.975 | 0.959 | 0.943 | 0.992 | 0.924 | 0.941 | 0.992 | 0.975 | 0.967 | 0.919 |
| 3jcs3 | 0.897 | 0.812 | 0.641 | 0.853 | 0.785 | 0.771 | 0.759 | 0.704 | 0.716 | 0.829 | 0.691 | 0.618 |
| 6az34 | 0.940 | 0.929 | 0.891 | 0.876 | 0.941 | 0.922 | 0.900 | 0.889 | 0.949 | 0.922 | 0.874 | 0.876 |
| 6j6gD | 0.936 | 0.946 | 0.851 | 0.905 | 0.911 | 0.935 | 0.870 | 0.921 | 0.889 | 0.935 | 0.884 | 0.814 |
| 3dilA | 0.976 | 0.984 | 0.952 | 0.917 | 0.952 | 0.942 | 0.936 | 0.952 | 0.984 | 0.959 | 0.984 | 0.959 |
| 5lj3Z | 0.989 | 0.989 | 0.894 | 0.918 | 0.943 | 0.977 | 0.944 | 0.955 | 0.955 | 0.977 | 0.929 | 0.966 |
| 5t2aE | 0.989 | 0.977 | 0.876 | 0.876 | 0.933 | 0.944 | 0.955 | 0.861 | 0.943 | 0.954 | 0.944 | 0.878 |
| 3p49A | 0.918 | 0.914 | 0.824 | 0.897 | 0.871 | 0.927 | 0.916 | 0.897 | 0.853 | 0.897 | 0.881 | 0.739 |
| 5wlcL2 | 0.894 | 0.875 | 0.708 | 0.783 | 0.840 | 0.875 | 0.824 | 0.863 | 0.870 | 0.857 | 0.778 | 0.556 |
| 6ufhA | 0.978 | 0.978 | 0.882 | 0.925 | 0.957 | 0.935 | 0.913 | 0.933 | 0.955 | 0.956 | 0.909 | 0.966 |
| 6qx91 | 0.952 | 0.964 | 0.843 | 0.941 | 0.927 | 0.964 | 0.914 | 0.878 | 0.900 | 0.964 | 0.940 | 0.937 |
| 6az37 | 0.980 | 1.000 | 0.863 | 0.873 | 0.923 | 0.980 | 0.917 | 0.889 | 0.936 | 0.939 | 0.863 | 0.913 |
| 6ft62 | 0.958 | 0.957 | 0.833 | 0.755 | 0.880 | 0.880 | 0.783 | 0.864 | 0.894 | 0.917 | 0.875 | 0.780 |
| 3pdrA | 0.969 | 0.958 | 0.907 | 0.889 | 0.891 | 0.917 | 0.896 | 0.917 | 0.947 | 0.926 | 0.880 | 0.917 |
| 4gxyA | 0.942 | 0.923 | 0.849 | 0.922 | 0.913 | 0.878 | 0.871 | 0.880 | 0.839 | 0.909 | 0.857 | 0.780 |
| 6oleD | 0.977 | 0.977 | 0.844 | 0.870 | 0.894 | 0.977 | 0.857 | 0.905 | 0.976 | 0.955 | 0.930 | 0.976 |
| 5xxb4 | 0.952 | 0.952 | 0.818 | 0.708 | 0.889 | 0.884 | 0.829 | 0.850 | 0.905 | 0.905 | 0.905 | 0.865 |
| 1u9sA | 0.958 | 0.969 | 0.898 | 0.918 | 0.959 | 0.959 | 0.926 | 0.903 | 0.949 | 0.958 | 0.926 | 0.958 |
| 5xy34 | 0.913 | 0.894 | 0.775 | 0.833 | 0.816 | 0.851 | 0.808 | 0.808 | 0.844 | 0.870 | 0.902 | 0.750 |
| 4v8pB2 | 0.950 | 0.950 | 0.809 | 0.711 | 0.809 | 0.900 | 0.821 | 0.895 | 0.895 | 0.923 | 0.857 | 0.865 |
| 3j79C | 1.000 | 0.981 | 0.889 | 0.857 | 0.893 | 0.943 | 0.902 | 0.840 | 0.941 | 0.926 | 0.941 | 0.962 |
| 1nbsB | 0.952 | 0.964 | 0.871 | 0.874 | 0.943 | 0.965 | 0.892 | 0.914 | 0.940 | 0.930 | 0.897 | 0.927 |
| 6jdvB | 1.000 | 1.000 | 0.966 | 0.907 | 0.966 | 0.977 | 0.977 | 0.977 | 0.989 | 0.989 | 1.000 | 0.977 |
| 6id0H | 0.889 | 0.889 | 0.821 | 0.821 | 0.889 | 0.842 | 0.800 | 0.800 | 0.842 | 0.865 | 0.821 | 0.857 |
| 3hhnC | 0.966 | 0.954 | 0.897 | 0.930 | 0.977 | 0.954 | 0.943 | 0.943 | 0.944 | 0.966 | 0.943 | 0.966 |
| 3ndbM | 0.957 | 0.897 | 0.854 | 0.854 | 0.897 | 0.828 | 0.844 | 0.874 | 0.847 | 0.881 | 0.848 | 0.835 |
| 6exn2 | 1.000 | 1.000 | 0.918 | 0.935 | 0.969 | 1.000 | 0.954 | 0.952 | 0.968 | 1.000 | 0.918 | 0.968 |
| 6ahdI | 0.982 | 1.000 | 0.863 | 0.808 | 0.982 | 0.982 | 0.963 | 0.963 | 0.964 | 0.964 | 0.929 | 0.920 |
| 3l3cS | 0.865 | 0.873 | 0.784 | 0.833 | 0.865 | 0.870 | 0.861 | 0.833 | 0.892 | 0.873 | 0.800 | 0.800 |
| 4uykR | 0.989 | 1.000 | 0.935 | 0.957 | 0.946 | 0.989 | 0.966 | 0.977 | 1.000 | 0.989 | 0.978 | 1.000 |
| 2il9A | 0.667 | 0.694 | 0.597 | 0.627 | 0.667 | 0.723 | 0.640 | 0.640 | 0.714 | 0.727 | 0.627 | 0.593 |
| 6n5qA | 0.971 | 0.971 | 0.877 | 0.943 | 0.943 | 0.930 | 0.889 | 0.895 | 0.901 | 0.957 | 0.904 | 0.957 |
| 3v7eC | 0.956 | 0.956 | 0.933 | 0.956 | 0.956 | 0.933 | 0.956 | 0.913 | 0.923 | 0.956 | 0.945 | 0.918 |
| 5ganV | 0.923 | 0.947 | 0.923 | 0.895 | 0.750 | 0.947 | 0.919 | 0.778 | 0.895 | 0.900 | 0.809 | 0.947 |
| 2z75B | 1.000 | 1.000 | 0.969 | 0.969 | 0.952 | 0.985 | 0.952 | 0.952 | 0.985 | 1.000 | 0.970 | 1.000 |
| 5zluX | 0.958 | 0.943 | 0.800 | 0.845 | 0.901 | 0.866 | 0.912 | 0.866 | 0.862 | 0.927 | 0.927 | 0.724 |
| 1vq89 | 0.955 | 0.970 | 0.899 | 0.909 | 0.925 | 0.923 | 0.927 | 0.892 | 0.938 | 0.939 | 0.925 | 0.925 |
| 5dm6Y | 0.986 | 0.986 | 0.943 | 0.986 | 0.971 | 0.971 | 0.955 | 0.923 | 0.923 | 0.957 | 0.986 | 0.939 |
| 6t4qC4 | 0.972 | 0.958 | 0.946 | 0.889 | 0.946 | 0.944 | 0.882 | 0.882 | 0.927 | 0.917 | 0.917 | 0.930 |
| 5mmiB | 0.973 | 0.959 | 0.853 | 0.921 | 0.919 | 0.959 | 0.946 | 0.944 | 0.944 | 0.931 | 0.959 | 0.899 |
| 4ybbDB | 0.986 | 0.971 | 0.919 | 0.986 | 0.972 | 0.971 | 0.941 | 0.909 | 0.909 | 0.957 | 0.958 | 0.941 |
| 6ek0L7 | 0.943 | 0.927 | 0.800 | 0.829 | 0.931 | 0.895 | 0.912 | 0.882 | 0.831 | 0.912 | 0.889 | 0.895 |
| 1nbsA | 0.952 | 0.968 | 0.892 | 0.875 | 0.985 | 0.954 | 0.875 | 0.933 | 0.951 | 0.938 | 0.954 | 0.935 |
| 6qdv2 | 0.971 | 1.000 | 0.882 | 0.919 | 1.000 | 1.000 | 0.973 | 1.000 | 1.000 | 1.000 | 1.000 | 0.909 |
| 4v8pB3 | 0.943 | 0.927 | 0.822 | 0.882 | 0.866 | 0.901 | 0.899 | 0.895 | 0.895 | 0.912 | 0.873 | 0.895 |
| 3jcs8 | 0.836 | 0.821 | 0.800 | 0.712 | 0.815 | 0.815 | 0.815 | 0.739 | 0.830 | 0.720 | 0.821 | 0.667 |
| 4kqyA | 0.975 | 0.975 | 0.897 | 0.963 | 0.950 | 0.949 | 0.949 | 0.935 | 0.937 | 0.962 | 0.950 | 0.889 |
| 6uesA | 0.930 | 0.952 | 0.892 | 0.892 | 0.918 | 0.918 | 0.930 | 0.940 | 0.964 | 0.927 | 0.941 | 0.927 |

| | | | | | | | | | | | | |
|---|---|---|---|---|---|---|---|---|---|---|---|---|
| 5xxb3 | 0.944 | 0.930 | 0.827 | 0.842 | 0.853 | 0.836 | 0.870 | 0.831 | 0.836 | 0.866 | 0.917 | 0.733 |
| 3j79B | 0.986 | 0.957 | 0.914 | 0.912 | 0.944 | 0.943 | 0.912 | 0.882 | 0.925 | 0.941 | 0.930 | 0.941 |
| 5o60B | 0.985 | 0.954 | 0.849 | 0.954 | 0.906 | 0.969 | 0.952 | 0.918 | 0.952 | 0.938 | 0.985 | 0.969 |
| 6rm3L70 | 0.927 | 0.927 | 0.746 | 0.829 | 0.886 | 0.912 | 0.895 | 0.866 | 0.895 | 0.927 | 0.886 | 0.912 |
| 6xyw3 | 0.840 | 0.816 | 0.750 | 0.741 | 0.741 | 0.769 | 0.833 | 0.766 | 0.826 | 0.826 | 0.840 | 0.826 |
| 4qlnA | 0.987 | 0.987 | 0.959 | 0.973 | 0.931 | 0.987 | 0.987 | 0.901 | 0.959 | 0.987 | 0.987 | 0.973 |
| 5xy33 | 0.918 | 0.881 | 0.689 | 0.820 | 0.848 | 0.852 | 0.852 | 0.815 | 0.830 | 0.842 | 0.833 | 0.708 |
| 4qk9A | 0.987 | 0.987 | 0.904 | 0.931 | 0.987 | 0.987 | 0.944 | 0.914 | 0.944 | 0.987 | 0.947 | 0.973 |
| 6s0xB | 0.881 | 0.881 | 0.833 | 0.862 | 0.842 | 0.862 | 0.897 | 0.893 | 0.877 | 0.881 | 0.897 | 0.915 |
| 5wtiB | 0.935 | 0.935 | 0.849 | 0.875 | 0.875 | 0.935 | 0.900 | 0.903 | 0.918 | 0.906 | 0.892 | 0.933 |
| 6v3aB | 0.933 | 0.900 | 0.844 | 0.806 | 0.867 | 0.931 | 0.900 | 0.848 | 0.909 | 0.949 | 0.918 | 0.846 |
| 5f9rA | 0.933 | 0.915 | 0.867 | 0.853 | 0.933 | 0.933 | 0.933 | 0.900 | 0.897 | 0.931 | 0.881 | 0.800 |
| 6ahdB | 0.966 | 0.966 | 0.949 | 0.931 | 0.897 | 0.912 | 0.912 | 0.853 | 0.912 | 0.947 | 0.912 | 0.929 |
| 5t5hD | 0.938 | 0.938 | 0.831 | 0.849 | 0.862 | 0.889 | 0.871 | 0.833 | 0.857 | 0.889 | 0.857 | 0.489 |
| 6ha1B | 0.951 | 0.951 | 0.794 | 0.794 | 0.935 | 0.918 | 0.949 | 0.967 | 0.881 | 0.915 | 0.933 | 0.918 |
| 5vt0R | 0.951 | 0.951 | 0.938 | 0.892 | 0.906 | 0.935 | 0.921 | 0.915 | 0.933 | 0.935 | 0.923 | 0.915 |
| 6ol3C | 1.000 | 1.000 | 0.945 | 0.967 | 1.000 | 0.989 | 0.989 | 0.989 | 1.000 | 1.000 | 0.989 | 0.978 |
| 3jb9P | 0.857 | 0.857 | 0.818 | 0.857 | 0.857 | 0.857 | 0.947 | 0.857 | 0.842 | 0.900 | 0.947 | 0.800 |
| 6ny2B | 0.968 | 0.935 | 0.825 | 0.918 | 0.952 | 0.951 | 0.935 | 0.968 | 0.951 | 0.952 | 0.933 | 0.968 |
| 6ck5A | 0.943 | 0.927 | 0.941 | 0.824 | 0.925 | 0.927 | 0.886 | 0.899 | 0.909 | 0.925 | 0.927 | 0.909 |
| 3f2qX | 0.969 | 0.952 | 0.952 | 0.939 | 0.938 | 0.923 | 0.906 | 0.921 | 0.921 | 0.939 | 0.889 | 1.000 |
| 4y1mB | 0.986 | 0.986 | 0.971 | 0.944 | 0.972 | 0.972 | 0.930 | 0.931 | 0.986 | 0.959 | 0.946 | 1.000 |
| 6dlrA | 0.952 | 0.952 | 0.892 | 0.906 | 0.952 | 0.938 | 0.938 | 0.952 | 0.968 | 0.921 | 0.906 | 0.938 |
| 6eriAB | 0.980 | 0.980 | 0.939 | 0.939 | 0.939 | 0.958 | 0.936 | 0.917 | 0.933 | 0.936 | 0.958 | 0.889 |
| 4wflA | 1.000 | 0.986 | 0.912 | 0.914 | 0.986 | 0.986 | 0.971 | 0.971 | 1.000 | 1.000 | 0.957 | 0.986 |
| 3jb9C | 0.947 | 0.947 | 0.929 | 0.929 | 0.929 | 0.929 | 0.912 | 0.949 | 0.983 | 0.947 | 0.912 | 0.947 |
| 6j6gE | 0.970 | 0.970 | 0.941 | 0.941 | 0.938 | 0.970 | 0.938 | 0.938 | 0.938 | 0.914 | 0.944 | 0.938 |
| 4lckC | 0.983 | 0.983 | 0.862 | 0.949 | 0.983 | 0.967 | 0.935 | 0.966 | 0.931 | 0.933 | 0.951 | 0.909 |
| 4frnA | 0.967 | 0.949 | 0.915 | 0.918 | 0.915 | 0.949 | 0.931 | 0.931 | 0.912 | 0.951 | 0.848 | 0.912 |
| 2xxaF | 0.986 | 1.000 | 0.986 | 0.972 | 0.930 | 0.986 | 0.943 | 0.943 | 0.986 | 0.986 | 0.972 | 0.941 |
| 6mj0A | 0.949 | 0.931 | 0.933 | 0.867 | 0.862 | 0.877 | 0.807 | 0.807 | 0.877 | 0.931 | 0.871 | 0.947 |
| 4w90C | 0.966 | 0.947 | 0.828 | 0.915 | 0.931 | 0.912 | 0.929 | 0.909 | 0.873 | 0.912 | 0.931 | 0.760 |
| 5u33B | 0.964 | 0.964 | 0.889 | 0.846 | 0.946 | 0.926 | 0.885 | 0.885 | 0.902 | 0.926 | 0.946 | 0.833 |
| 6dmcA | 0.984 | 0.984 | 0.933 | 0.968 | 0.968 | 0.984 | 0.968 | 0.968 | 0.984 | 0.984 | 0.969 | 0.968 |
| 4y1jA | 0.985 | 0.985 | 0.985 | 0.970 | 0.970 | 0.985 | 0.969 | 0.954 | 0.969 | 0.969 | 0.985 | 1.000 |
| 3suhX | 0.951 | 0.933 | 0.871 | 0.903 | 0.933 | 0.933 | 0.915 | 0.918 | 0.967 | 0.967 | 0.867 | 0.929 |
| 4rzdA | 0.966 | 0.966 | 0.881 | 0.915 | 0.983 | 0.964 | 0.982 | 0.963 | 0.964 | 1.000 | 0.982 | 0.982 |
| 6n2vA | 0.984 | 0.984 | 1.000 | 0.954 | 0.984 | 0.984 | 0.938 | 0.984 | 0.952 | 0.984 | 0.968 | 0.984 |
| 6az35 | 0.980 | 0.980 | 0.863 | 0.885 | 0.960 | 0.939 | 0.939 | 0.958 | 0.958 | 0.939 | 1.000 | 0.980 |
| 6id1F | 0.923 | 0.917 | 0.960 | 0.929 | 0.923 | 0.917 | 0.880 | 0.880 | 0.917 | 0.880 | 1.000 | 0.870 |
| 4oquA | 0.985 | 0.985 | 0.954 | 0.954 | 0.985 | 0.985 | 0.985 | 0.952 | 0.951 | 0.969 | 0.954 | 0.968 |
| 6jxmB | 0.966 | 0.966 | 0.912 | 0.897 | 0.929 | 0.877 | 0.900 | 0.915 | 0.877 | 0.893 | 0.915 | 0.889 |
| 6jooB | 0.941 | 0.962 | 0.962 | 0.962 | 0.962 | 0.923 | 0.962 | 0.960 | 0.962 | 0.962 | 0.962 | 0.939 |
| 3ktwC | 0.967 | 0.967 | 0.889 | 0.951 | 0.949 | 0.949 | 0.933 | 0.903 | 0.951 | 0.967 | 0.885 | 0.784 |
| 6dvkH | 0.984 | 1.000 | 0.984 | 0.969 | 0.968 | 0.967 | 0.984 | 0.967 | 0.967 | 0.967 | 0.938 | 0.915 |
| 2yghA | 0.931 | 0.931 | 0.931 | 0.900 | 0.881 | 0.881 | 0.893 | 0.912 | 0.881 | 0.912 | 0.897 | 0.929 |
| 5ml7A | 0.946 | 0.946 | 0.828 | 0.912 | 0.912 | 0.931 | 0.946 | 0.946 | 0.926 | 0.893 | 0.931 | 0.863 |
| 5b2oB | 0.900 | 0.923 | 0.800 | 0.750 | 0.857 | 0.900 | 0.900 | 0.842 | 0.878 | 0.895 | 0.927 | 0.927 |

| | | | | | | | | | | | | |
|---|---|---|---|---|---|---|---|---|---|---|---|---|
| 5x2hB | 1.000 | 0.979 | 0.960 | 0.957 | 1.000 | 0.936 | 0.958 | 0.958 | 0.979 | 0.979 | 0.960 | 1.000 |
| 3iwnA | 0.946 | 0.946 | 0.926 | 0.929 | 0.964 | 0.946 | 0.906 | 0.889 | 0.946 | 0.926 | 0.909 | 0.963 |
| 1m5oB | 0.952 | 0.952 | 0.864 | 0.905 | 0.927 | 0.927 | 0.900 | 0.923 | 0.900 | 0.900 | 0.905 | 0.850 |
| 4rumA | 1.000 | 1.000 | 0.964 | 0.983 | 0.983 | 1.000 | 1.000 | 0.947 | 1.000 | 1.000 | 1.000 | 0.983 |
| 3culD | 1.000 | 0.983 | 0.931 | 0.900 | 0.966 | 0.983 | 0.983 | 0.983 | 0.983 | 0.966 | 0.983 | 0.926 |
| 6mwnA | 0.983 | 0.983 | 0.967 | 0.949 | 0.983 | 0.966 | 0.947 | 0.915 | 0.947 | 0.931 | 0.968 | 0.929 |
| 5t83A | 0.870 | 0.851 | 0.784 | 0.851 | 0.851 | 0.844 | 0.837 | 0.844 | 0.864 | 0.844 | 0.800 | 0.857 |
| 4lvwA | 0.952 | 0.967 | 0.935 | 0.885 | 0.968 | 0.967 | 0.967 | 0.967 | 0.967 | 0.967 | 0.968 | 1.000 |
| 5osg2 | 0.909 | 0.909 | 0.857 | 0.865 | 0.909 | 0.882 | 0.882 | 0.909 | 0.909 | 0.800 | 0.914 | 0.786 |
| 1wz2C | 0.958 | 0.958 | 0.898 | 0.898 | 0.939 | 0.917 | 0.840 | 0.917 | 0.936 | 0.917 | 0.846 | 0.939 |
| 3owiB | 0.980 | 0.980 | 0.902 | 0.941 | 0.902 | 0.939 | 0.898 | 0.917 | 0.902 | 0.960 | 0.920 | 0.960 |
| 4v8bAB | 0.889 | 0.889 | 0.844 | 0.826 | 0.909 | 0.909 | 0.864 | 0.851 | 0.909 | 0.833 | 0.773 | 0.688 |
| 3k0jE | 1.000 | 1.000 | 0.826 | 0.875 | 0.898 | 0.936 | 0.957 | 0.957 | 0.955 | 0.933 | 0.875 | 1.000 |
| 6c0f6 | 0.971 | 0.971 | 0.778 | 0.882 | 0.914 | 0.914 | 0.889 | 0.882 | 0.909 | 0.941 | 0.971 | 0.800 |
| 5aoxF | 0.967 | 0.967 | 0.949 | 0.933 | 0.949 | 0.967 | 0.931 | 0.912 | 0.949 | 0.967 | 0.933 | 0.966 |
| 3q1qC | 0.980 | 0.980 | 0.824 | 0.943 | 0.941 | 0.941 | 0.941 | 0.863 | 0.960 | 0.902 | 0.906 | 0.980 |
| 4v8dAB | 0.933 | 0.955 | 0.727 | 0.933 | 0.864 | 0.909 | 0.955 | 0.884 | 0.884 | 0.909 | 0.833 | 0.757 |
| 4mgnA | 0.955 | 0.930 | 0.894 | 0.933 | 0.933 | 0.930 | 0.844 | 0.909 | 0.930 | 0.909 | 0.870 | 0.878 |
| 5u3gB | 0.979 | 0.979 | 0.936 | 0.958 | 0.958 | 0.979 | 0.913 | 0.913 | 0.957 | 0.979 | 0.957 | 0.957 |
| 6b14R | 0.936 | 0.913 | 0.816 | 0.840 | 0.863 | 0.913 | 0.898 | 0.844 | 0.894 | 0.889 | 0.875 | 0.913 |
| 4yazA | 0.980 | 0.980 | 0.941 | 0.898 | 0.980 | 0.936 | 0.936 | 0.889 | 0.917 | 0.936 | 0.960 | 0.958 |
| 6jq5A | 0.973 | 0.973 | 0.821 | 0.889 | 0.973 | 0.973 | 0.919 | 0.944 | 0.944 | 0.919 | 0.895 | 0.973 |
| 3w3sB | 0.970 | 0.970 | 0.925 | 0.971 | 0.985 | 0.970 | 0.941 | 0.923 | 0.970 | 0.955 | 0.954 | 0.954 |
| 3am1B | 0.983 | 0.983 | 0.933 | 0.949 | 0.983 | 0.967 | 0.983 | 0.929 | 0.966 | 0.951 | 0.915 | 0.912 |
| 5lzdy | 0.985 | 0.985 | 0.925 | 0.970 | 0.985 | 0.985 | 0.941 | 0.970 | 0.985 | 0.971 | 0.955 | 0.985 |
| 3umyB | 0.952 | 0.952 | 0.913 | 0.870 | 0.898 | 0.955 | 0.894 | 0.930 | 0.952 | 0.889 | 0.909 | 0.930 |
| 6ah3T | 0.952 | 0.952 | 0.884 | 0.857 | 0.952 | 0.952 | 0.900 | 0.884 | 0.927 | 0.909 | 0.864 | 0.800 |
| 3adbC | 0.985 | 0.985 | 0.954 | 0.954 | 0.985 | 0.985 | 0.969 | 0.938 | 0.985 | 0.970 | 0.955 | 0.970 |
| 6cu1A | 0.982 | 0.963 | 0.963 | 0.929 | 0.964 | 0.964 | 0.946 | 0.912 | 0.963 | 0.982 | 0.929 | 0.963 |
| 5on2B | 0.962 | 0.962 | 0.962 | 0.926 | 0.980 | 0.960 | 0.941 | 0.840 | 0.920 | 0.941 | 0.980 | 0.962 |
| 3amtB | 0.977 | 0.952 | 0.930 | 0.909 | 0.952 | 0.952 | 0.930 | 0.930 | 0.930 | 0.952 | 0.909 | 0.927 |
| 6rjaD | 0.927 | 0.950 | 0.884 | 0.829 | 0.930 | 0.950 | 0.950 | 0.950 | 0.950 | 0.927 | 0.952 | 0.865 |
| 4v8nAW | 0.714 | 0.714 | 0.516 | 0.606 | 0.667 | 0.643 | 0.516 | 0.690 | 0.640 | 0.692 | 0.500 | 0.778 |
| 6v5bD | 0.946 | 0.963 | 0.964 | 0.963 | 0.963 | 0.963 | 0.946 | 0.946 | 0.963 | 0.963 | 0.946 | 0.923 |
| 5vppQV | 0.895 | 0.895 | 0.700 | 0.800 | 0.872 | 0.872 | 0.865 | 0.865 | 0.824 | 0.833 | 0.895 | 0.800 |
| 6uggA | 0.977 | 0.977 | 0.909 | 0.930 | 0.955 | 0.977 | 0.977 | 0.870 | 0.955 | 0.933 | 0.955 | 0.977 |
| 3d2vA | 0.974 | 0.974 | 0.950 | 0.974 | 0.900 | 0.974 | 0.895 | 0.895 | 0.950 | 0.923 | 0.872 | 0.974 |
| 5ccbN | 0.978 | 0.955 | 0.791 | 0.930 | 0.930 | 0.913 | 0.930 | 0.909 | 0.909 | 0.930 | 0.889 | 0.930 |
| 6svsA | 1.000 | 1.000 | 0.898 | 0.958 | 1.000 | 0.979 | 0.936 | 0.957 | 0.889 | 0.979 | 0.979 | 0.957 |
| 5j8bx | 0.976 | 0.976 | 0.950 | 0.950 | 0.952 | 0.976 | 0.909 | 0.927 | 0.976 | 0.927 | 0.909 | 0.950 |
| 2zzmB | 0.950 | 0.950 | 0.769 | 0.791 | 0.927 | 0.837 | 0.884 | 0.905 | 0.850 | 0.927 | 0.905 | 0.889 |
| 6ufmA | 0.977 | 0.977 | 0.930 | 0.955 | 0.977 | 0.977 | 0.955 | 0.889 | 0.977 | 0.955 | 0.955 | 0.955 |
| 6v3av | 1.000 | 1.000 | 0.878 | 0.950 | 1.000 | 1.000 | 1.000 | 0.974 | 0.976 | 1.000 | 0.952 | 1.000 |
| 1h3eB | 0.980 | 0.980 | 0.923 | 0.960 | 0.980 | 0.941 | 0.980 | 0.902 | 0.960 | 0.980 | 0.923 | 0.958 |
| 6i0yV | 0.952 | 0.952 | 0.829 | 0.878 | 0.857 | 0.780 | 0.800 | 0.811 | 0.737 | 0.842 | 0.850 | 0.667 |
| 1j2bC | 0.947 | 0.947 | 0.829 | 0.923 | 0.950 | 0.947 | 0.947 | 0.923 | 0.947 | 0.947 | 0.900 | 0.919 |
| 5o2rx | 1.000 | 1.000 | 0.909 | 0.930 | 0.976 | 0.976 | 1.000 | 0.930 | 1.000 | 0.976 | 0.977 | 0.895 |
| 3a2kC | 0.977 | 0.977 | 0.744 | 0.844 | 0.930 | 0.955 | 0.930 | 0.909 | 0.977 | 0.955 | 0.955 | 0.977 |

| | | | | | | | | | | | | |
|---|---|---|---|---|---|---|---|---|---|---|---|---|
| 6q977 | 0.947 | 0.947 | 0.800 | 0.889 | 0.743 | 0.919 | 0.919 | 0.800 | 0.974 | 0.833 | 0.829 | 0.923 |
| 5ah5D | 0.978 | 0.978 | 0.957 | 0.913 | 0.957 | 0.978 | 0.978 | 0.870 | 0.955 | 0.957 | 0.917 | 0.978 |
| 4jf2A | 0.960 | 0.958 | 0.920 | 0.939 | 0.960 | 0.958 | 0.939 | 0.958 | 0.979 | 0.980 | 0.960 | 0.960 |
| 6t4q6 | 0.976 | 0.976 | 0.905 | 0.905 | 0.927 | 0.930 | 0.923 | 0.927 | 0.976 | 0.976 | 0.884 | 0.976 |
| 6az12 | 0.833 | 0.800 | 0.737 | 0.789 | 0.743 | 0.800 | 0.743 | 0.722 | 0.812 | 0.765 | 0.811 | 0.560 |
| 1qf6B | 0.976 | 0.976 | 0.878 | 0.930 | 0.976 | 0.976 | 0.909 | 0.905 | 0.952 | 0.930 | 0.884 | 0.976 |
| 6r5q2 | 0.824 | 0.824 | 0.727 | 0.824 | 0.800 | 0.800 | 0.800 | 0.722 | 0.667 | 0.733 | 0.778 | 0.476 |
| 2iy5T | 0.977 | 0.927 | 0.718 | 0.809 | 0.878 | 0.905 | 0.952 | 0.952 | 0.955 | 0.905 | 0.800 | 0.952 |
| 4v5lAY | 0.977 | 0.977 | 0.955 | 0.818 | 0.955 | 0.933 | 0.952 | 0.884 | 0.955 | 0.955 | 0.870 | 0.872 |
| 5el63K | 0.857 | 0.857 | 0.625 | 0.645 | 0.800 | 0.759 | 0.800 | 0.828 | 0.667 | 0.692 | 0.774 | 0.545 |
| 6t7t6 | 0.955 | 0.930 | 0.889 | 0.864 | 0.933 | 0.955 | 0.909 | 0.909 | 0.977 | 0.977 | 0.889 | 0.952 |
| 4wj4B | 0.977 | 0.977 | 0.864 | 0.905 | 0.977 | 0.930 | 0.977 | 0.930 | 0.977 | 0.955 | 0.913 | 0.977 |
| 5lzsii | 0.947 | 0.947 | 0.757 | 0.750 | 0.789 | 0.842 | 0.850 | 0.919 | 0.919 | 0.882 | 0.750 | 0.909 |
| 6r87B | 0.895 | 0.895 | 0.850 | 0.842 | 0.842 | 0.923 | 0.850 | 0.842 | 0.833 | 0.865 | 0.750 | 0.800 |
| 4v5gAY | 0.706 | 0.706 | 0.743 | 0.686 | 0.686 | 0.686 | 0.667 | 0.629 | 0.647 | 0.706 | 0.632 | 0.300 |
| 6q9a7 | 0.889 | 0.923 | 0.706 | 0.710 | 0.846 | 0.828 | 0.828 | 0.889 | 0.923 | 0.857 | 0.857 | 0.833 |
| 2zueB | 0.977 | 0.977 | 0.933 | 0.933 | 0.809 | 0.955 | 0.930 | 0.909 | 0.955 | 0.977 | 0.913 | 0.977 |
| 1g59B | 0.977 | 0.977 | 0.930 | 0.952 | 0.905 | 0.977 | 0.909 | 0.857 | 0.884 | 0.952 | 0.909 | 0.952 |
| 4rdxC | 0.952 | 0.952 | 0.878 | 0.905 | 0.909 | 0.930 | 0.930 | 0.864 | 0.952 | 0.976 | 0.952 | 0.950 |
| 5x6bP | 0.927 | 0.950 | 0.878 | 0.905 | 0.900 | 0.947 | 0.947 | 0.895 | 0.974 | 0.927 | 0.900 | 0.919 |
| 1il2C | 0.952 | 0.952 | 0.884 | 0.930 | 0.952 | 0.884 | 0.930 | 0.844 | 0.837 | 0.930 | 0.884 | 0.927 |
| 6t4q7 | 0.974 | 0.974 | 0.895 | 0.842 | 0.974 | 0.905 | 0.950 | 0.900 | 0.950 | 0.905 | 0.878 | 0.919 |
| 2csxC | 0.976 | 0.976 | 0.927 | 0.952 | 0.952 | 0.976 | 0.930 | 0.930 | 0.976 | 0.976 | 0.930 | 0.976 |
| 6tbvPTR1 | 1.000 | 1.000 | 1.000 | 0.952 | 1.000 | 0.977 | 0.977 | 0.930 | 0.952 | 0.977 | 0.955 | 1.000 |
| 6tb3n | 0.977 | 0.977 | 0.909 | 0.955 | 0.955 | 0.955 | 0.955 | 0.930 | 0.977 | 0.955 | 0.930 | 0.977 |
| 6qdwv | 0.615 | 0.667 | 0.133 | 0.333 | 0.500 | 0.364 | 0.545 | 0.222 | 0.600 | 0.444 | 0.375 | 0.000 |
| 1gaxC | 0.977 | 0.977 | 0.952 | 0.864 | 0.930 | 0.977 | 0.955 | 0.955 | 0.977 | 0.955 | 0.889 | 0.977 |
| 6ek0S6 | 0.769 | 0.769 | 0.562 | 0.667 | 0.759 | 0.769 | 0.593 | 0.667 | 0.750 | 0.720 | 0.667 | 0.750 |
| 2dr2B | 0.950 | 0.895 | 0.923 | 0.900 | 0.919 | 0.872 | 0.905 | 0.950 | 0.895 | 0.872 | 0.864 | 0.878 |
| 4v7lAY | 0.765 | 0.765 | 0.686 | 0.647 | 0.703 | 0.765 | 0.722 | 0.722 | 0.839 | 0.765 | 0.706 | 0.800 |
| 4lckB | 0.977 | 0.977 | 0.930 | 0.977 | 0.955 | 0.977 | 0.952 | 0.864 | 0.955 | 0.955 | 0.955 | 0.977 |
| 3wqyC | 0.957 | 0.978 | 0.884 | 0.909 | 0.957 | 0.978 | 0.936 | 0.957 | 0.978 | 0.978 | 0.917 | 0.957 |
| 4v9iAY | 0.667 | 0.667 | 0.556 | 0.500 | 0.571 | 0.429 | 0.533 | 0.471 | 0.714 | 0.714 | 0.500 | 0.800 |
| 6ip5zu | 0.927 | 0.950 | 0.900 | 0.837 | 0.927 | 0.905 | 0.884 | 0.927 | 0.950 | 0.905 | 0.864 | 0.950 |
| 1j1uB | 0.955 | 0.955 | 0.857 | 0.952 | 0.952 | 0.977 | 0.913 | 0.930 | 0.977 | 0.977 | 0.894 | 0.927 |
| 3rg5A | 0.966 | 0.947 | 0.881 | 0.828 | 0.947 | 0.947 | 0.912 | 0.912 | 0.929 | 0.931 | 0.857 | 0.929 |
| 1u0bA | 0.976 | 0.976 | 0.905 | 0.952 | 0.976 | 0.952 | 0.952 | 0.950 | 1.000 | 0.930 | 0.864 | 1.000 |
| 4yyeC | 0.976 | 0.976 | 0.773 | 0.927 | 0.905 | 0.952 | 0.930 | 0.905 | 0.952 | 0.976 | 0.889 | 0.950 |
| 4prfB | 1.000 | 1.000 | 0.884 | 0.977 | 1.000 | 1.000 | 1.000 | 0.933 | 1.000 | 1.000 | 1.000 | 1.000 |
| 3q3zA | 0.958 | 0.936 | 0.870 | 0.833 | 0.917 | 0.894 | 0.870 | 0.875 | 0.913 | 0.913 | 0.898 | 0.936 |
| 5d8hA | 0.941 | 0.882 | 0.800 | 0.800 | 0.857 | 0.882 | 0.833 | 0.849 | 0.882 | 0.857 | 0.941 | 0.938 |
| 3akzE | 0.977 | 0.977 | 0.909 | 0.955 | 0.955 | 0.909 | 0.905 | 0.927 | 0.977 | 0.977 | 0.933 | 0.933 |
| 2d6fF | 0.977 | 0.977 | 0.909 | 0.844 | 0.955 | 0.977 | 0.952 | 0.909 | 0.977 | 0.977 | 0.894 | 0.952 |
| 3j7a7 | 0.889 | 0.750 | 0.545 | 0.571 | 0.571 | 0.667 | 0.500 | 0.706 | 0.667 | 0.667 | 0.471 | 0.462 |
| 2dlcY | 0.914 | 0.914 | 0.865 | 0.865 | 0.778 | 0.889 | 0.914 | 0.833 | 0.889 | 0.865 | 0.865 | 0.941 |
| 5czzB | 1.000 | 1.000 | 0.938 | 0.938 | 1.000 | 0.970 | 1.000 | 0.968 | 1.000 | 1.000 | 1.000 | 1.000 |
| 6cae1y | 0.952 | 0.952 | 0.878 | 0.884 | 0.930 | 0.927 | 0.950 | 0.878 | 0.923 | 0.952 | 0.905 | 0.865 |
| 5e6mC | 0.976 | 0.976 | 0.878 | 0.829 | 0.976 | 0.930 | 0.905 | 0.884 | 0.952 | 0.909 | 0.837 | 0.976 |

| | | | | | | | | | | | | |
|---|---|---|---|---|---|---|---|---|---|---|---|---|
| 4znpA | 1.000 | 0.976 | 0.955 | 0.955 | 0.952 | 0.930 | 0.976 | 0.976 | 0.976 | 0.952 | 0.977 | 0.865 |
| 2derC | 0.944 | 0.914 | 0.732 | 0.821 | 0.800 | 0.706 | 0.800 | 0.757 | 0.849 | 0.778 | 0.718 | 0.714 |
| 5wt3C | 0.978 | 0.978 | 0.864 | 0.864 | 0.955 | 0.955 | 0.913 | 0.909 | 0.933 | 0.955 | 0.870 | 0.955 |
| 5ud5D | 0.979 | 0.957 | 0.844 | 0.933 | 0.870 | 0.957 | 0.957 | 0.917 | 0.936 | 0.936 | 0.957 | 0.979 |
| 5wwrC | 0.905 | 0.905 | 0.829 | 0.857 | 0.909 | 0.909 | 0.884 | 0.864 | 0.905 | 0.909 | 0.864 | 0.900 |
| 2zniC | 0.927 | 0.927 | 0.905 | 0.900 | 0.872 | 0.878 | 0.927 | 0.905 | 0.927 | 0.927 | 0.857 | 0.872 |
| 6lxdD | 0.826 | 0.826 | 0.723 | 0.766 | 0.936 | 0.851 | 0.851 | 0.844 | 0.773 | 0.800 | 0.833 | 0.667 |
| 3la5A | 0.936 | 0.936 | 0.889 | 0.894 | 0.917 | 0.913 | 0.913 | 0.837 | 0.913 | 0.913 | 0.844 | 0.913 |
| 5kpyA | 0.947 | 0.947 | 0.889 | 0.800 | 0.900 | 0.947 | 0.927 | 0.789 | 0.919 | 0.878 | 0.837 | 0.947 |
| 6az36 | 0.778 | 0.824 | 0.842 | 0.625 | 0.778 | 0.778 | 0.842 | 0.706 | 0.706 | 0.706 | 0.737 | 0.778 |
| 2du3D | 0.927 | 0.927 | 0.878 | 0.829 | 0.905 | 0.927 | 0.878 | 0.809 | 0.895 | 0.950 | 0.809 | 0.919 |
| 2oiuP | 0.978 | 0.978 | 0.933 | 0.978 | 0.978 | 0.978 | 0.978 | 0.978 | 0.978 | 0.978 | 0.978 | 0.978 |
| 5tpyA | 0.980 | 0.980 | 0.917 | 0.833 | 0.898 | 0.980 | 0.917 | 0.939 | 0.939 | 0.980 | 0.960 | 0.958 |
| 6gazAV | 1.000 | 0.971 | 0.857 | 0.857 | 0.971 | 1.000 | 0.973 | 0.919 | 0.944 | 1.000 | 0.872 | 0.909 |
| 1kxkA | 1.000 | 1.000 | 0.979 | 0.979 | 0.955 | 1.000 | 0.955 | 0.930 | 0.930 | 1.000 | 0.894 | 1.000 |
| 5ob3A | 0.976 | 0.950 | 0.950 | 0.857 | 0.930 | 0.976 | 0.976 | 0.927 | 0.878 | 0.950 | 0.927 | 0.900 |
| 3ivnA | 0.913 | 0.936 | 0.875 | 0.840 | 0.889 | 0.889 | 0.889 | 0.884 | 0.889 | 0.913 | 0.833 | 0.909 |
| 3ephE | 0.842 | 0.842 | 0.683 | 0.821 | 0.780 | 0.821 | 0.821 | 0.821 | 0.865 | 0.800 | 0.634 | 0.865 |
| 6u8dA | 1.000 | 0.981 | 0.962 | 0.906 | 0.943 | 0.981 | 1.000 | 0.962 | 1.000 | 0.981 | 1.000 | 1.000 |
| 2qusA | 0.952 | 0.952 | 0.927 | 0.952 | 0.952 | 0.952 | 0.930 | 0.900 | 0.952 | 0.952 | 0.930 | 0.952 |
| 4pqvA | 0.976 | 0.950 | 0.927 | 0.900 | 0.927 | 0.895 | 0.865 | 0.865 | 0.872 | 0.895 | 0.950 | 0.833 |
| 6p2hA | 0.974 | 0.974 | 0.947 | 0.950 | 0.947 | 0.947 | 0.919 | 0.889 | 0.947 | 0.950 | 0.900 | 0.974 |
| 4wzjV | 1.000 | 1.000 | 0.905 | 0.909 | 0.977 | 1.000 | 0.952 | 0.952 | 1.000 | 1.000 | 0.977 | 0.976 |
| 4fe5B | 0.955 | 0.978 | 0.818 | 0.933 | 0.913 | 0.884 | 0.818 | 0.850 | 0.955 | 0.978 | 0.864 | 0.909 |
| 3skiB | 0.958 | 0.936 | 0.833 | 0.917 | 0.894 | 0.894 | 0.917 | 0.913 | 0.958 | 0.936 | 0.913 | 0.958 |
| 6gawBB | 1.000 | 1.000 | 0.811 | 0.882 | 0.971 | 0.973 | 0.973 | 0.973 | 0.923 | 0.973 | 0.971 | 0.944 |
| 5a8lB | 0.737 | 0.909 | 0.571 | 0.571 | 0.783 | 0.909 | 0.857 | 0.800 | 0.857 | 0.818 | 0.778 | 0.667 |
| 1h4sT | 0.974 | 0.974 | 0.923 | 0.974 | 0.895 | 0.829 | 0.900 | 0.950 | 0.947 | 0.923 | 0.974 | 0.974 |
| 3r4fA | 1.000 | 1.000 | 0.933 | 1.000 | 0.978 | 0.978 | 0.978 | 0.978 | 0.978 | 0.957 | 0.957 | 1.000 |
| 3egzB | 1.000 | 1.000 | 0.944 | 0.971 | 0.970 | 0.970 | 0.970 | 1.000 | 0.970 | 1.000 | 1.000 | 1.000 |
| 4xwfA | 0.973 | 0.973 | 0.923 | 0.947 | 0.919 | 0.973 | 0.973 | 0.973 | 0.973 | 0.973 | 0.973 | 0.973 |
| 3nkbB | 1.000 | 0.973 | 0.850 | 0.947 | 0.923 | 0.973 | 0.973 | 0.947 | 0.973 | 0.919 | 0.865 | 1.000 |
| 5b63D | 0.941 | 0.941 | 0.824 | 0.857 | 0.914 | 0.857 | 0.889 | 0.882 | 0.875 | 0.824 | 0.889 | 0.759 |
| 6rflU | 0.971 | 0.971 | 0.944 | 0.971 | 0.944 | 0.971 | 0.944 | 0.895 | 0.919 | 0.919 | 0.872 | 0.971 |
| 5t5aA | 0.933 | 0.957 | 0.864 | 0.979 | 0.917 | 0.933 | 0.913 | 0.913 | 0.957 | 0.913 | 0.936 | 0.955 |
| 5btpA | 1.000 | 0.974 | 0.842 | 1.000 | 0.950 | 0.950 | 0.974 | 0.974 | 0.974 | 0.927 | 0.976 | 0.974 |
| 2czjB | 0.944 | 0.944 | 0.944 | 0.857 | 0.914 | 0.857 | 0.833 | 0.889 | 0.909 | 0.882 | 0.684 | 0.667 |
| 3icqD | 0.933 | 0.933 | 0.688 | 0.875 | 0.903 | 0.839 | 0.903 | 0.867 | 0.933 | 0.933 | 0.938 | 0.897 |
| 2hvyE | 1.000 | 1.000 | 0.938 | 1.000 | 0.970 | 1.000 | 0.970 | 0.941 | 1.000 | 0.970 | 1.000 | 1.000 |
| 5ddpA | 0.970 | 0.970 | 0.938 | 0.941 | 0.970 | 0.970 | 0.938 | 0.938 | 0.938 | 0.970 | 0.970 | 0.970 |
| 1un6E | 0.947 | 0.973 | 0.914 | 0.914 | 0.944 | 0.947 | 0.973 | 0.944 | 0.889 | 0.944 | 0.944 | 0.882 |
| 6db8R | 0.944 | 0.944 | 0.842 | 0.889 | 0.944 | 0.944 | 0.914 | 0.914 | 0.944 | 0.944 | 0.944 | 0.914 |
| 3rw6F | 1.000 | 1.000 | 0.947 | 0.950 | 0.974 | 0.947 | 0.973 | 0.973 | 0.947 | 1.000 | 0.974 | 0.973 |
| 6pmoA | 0.950 | 0.950 | 0.923 | 0.923 | 0.950 | 0.950 | 0.950 | 0.950 | 0.950 | 0.950 | 0.950 | 0.950 |
| 4m4oB | 0.919 | 0.919 | 0.919 | 0.865 | 0.865 | 0.865 | 0.889 | 0.889 | 0.889 | 0.919 | 0.821 | 0.857 |
| 3lwrD | 1.000 | 1.000 | 0.938 | 1.000 | 1.000 | 1.000 | 1.000 | 0.968 | 0.968 | 1.000 | 0.970 | 1.000 |
| 4v2sQ | 0.971 | 0.971 | 1.000 | 0.944 | 0.971 | 0.971 | 0.971 | 0.941 | 0.971 | 0.971 | 0.914 | 0.800 |
| 1dk1B | 0.973 | 0.944 | 0.889 | 0.973 | 0.919 | 0.944 | 0.919 | 0.889 | 0.944 | 0.919 | 0.919 | 0.919 |

| | | | | | | | | | | | | |
|---|---|---|---|---|---|---|---|---|---|---|---|---|
| 3j7yB | 0.971 | 0.971 | 0.944 | 0.971 | 1.000 | 0.971 | 0.941 | 0.971 | 0.944 | 1.000 | 0.944 | 0.941 |
| 4rgeA | 0.974 | 0.947 | 0.923 | 0.905 | 0.895 | 0.889 | 0.923 | 0.974 | 0.919 | 0.919 | 0.895 | 0.974 |
| 1serT | 0.947 | 0.974 | 0.927 | 0.923 | 0.974 | 0.974 | 0.950 | 0.878 | 0.950 | 0.974 | 0.927 | 0.947 |
| 2nreF | 0.933 | 0.867 | 0.867 | 0.741 | 0.903 | 0.839 | 0.897 | 0.839 | 0.769 | 0.786 | 0.875 | 0.696 |
| 5lyuA | 0.976 | 0.976 | 0.976 | 0.976 | 0.976 | 0.976 | 0.950 | 0.927 | 0.976 | 0.976 | 0.895 | 0.900 |
| 4k27U | 1.000 | 1.000 | 0.970 | 0.971 | 1.000 | 1.000 | 1.000 | 1.000 | 1.000 | 1.000 | 0.909 | 1.000 |
| 1mzpB | 0.923 | 0.923 | 0.929 | 0.897 | 0.867 | 0.929 | 0.857 | 0.923 | 0.923 | 0.889 | 0.867 | 0.880 |
| 6laxA | 0.970 | 1.000 | 0.812 | 0.933 | 0.968 | 0.968 | 0.968 | 0.968 | 0.968 | 0.938 | 0.909 | 0.903 |
| 5uq8x | 0.933 | 0.857 | 0.857 | 0.824 | 0.933 | 0.933 | 0.769 | 0.667 | 0.857 | 0.933 | 0.800 | 0.833 |
| 4pkdV | 0.968 | 0.968 | 1.000 | 0.968 | 0.968 | 0.968 | 0.933 | 0.903 | 0.897 | 0.968 | 0.968 | 0.966 |
| 2fk6R | 0.970 | 0.970 | 0.938 | 0.903 | 0.938 | 0.970 | 0.970 | 0.941 | 0.970 | 0.938 | 0.941 | 0.938 |
| 3e5cA | 1.000 | 1.000 | 0.968 | 0.968 | 0.938 | 0.968 | 0.938 | 0.968 | 0.968 | 0.968 | 0.938 | 1.000 |
| 4encA | 1.000 | 1.000 | 1.000 | 1.000 | 0.968 | 0.933 | 0.966 | 1.000 | 0.966 | 0.966 | 0.966 | 1.000 |
| 4ojiA | 0.973 | 0.944 | 0.914 | 0.944 | 0.857 | 0.914 | 0.944 | 0.973 | 0.944 | 0.889 | 0.889 | 0.973 |
| 5tf6D | 0.952 | 1.000 | 0.696 | 0.917 | 0.857 | 0.900 | 0.706 | 0.800 | 0.737 | 1.000 | 0.778 | 0.778 |
| 6aayB | 1.000 | 1.000 | 0.762 | 0.909 | 1.000 | 1.000 | 1.000 | 1.000 | 1.000 | 1.000 | 1.000 | 0.947 |
| 2qwyA | 0.960 | 0.917 | 0.889 | 0.846 | 0.880 | 0.917 | 0.846 | 0.750 | 0.833 | 0.870 | 0.923 | 0.818 |
| 6iv8B | 0.889 | 0.941 | 0.824 | 0.941 | 0.889 | 0.889 | 0.889 | 0.889 | 0.941 | 0.941 | 0.941 | 0.875 |
| 3npqA | 0.923 | 0.923 | 0.857 | 0.923 | 0.889 | 0.923 | 0.880 | 0.880 | 0.923 | 0.923 | 0.889 | 0.833 |
| 5y7mB | 0.917 | 0.917 | 0.870 | 0.783 | 0.917 | 0.870 | 0.870 | 0.800 | 0.818 | 0.870 | 0.917 | 0.818 |
| 5xwyB | 0.889 | 0.800 | 0.800 | 0.889 | 0.889 | 0.889 | 1.000 | 1.000 | 0.889 | 0.889 | 0.889 | 0.889 |
| 6e9eB | 1.000 | 1.000 | 0.444 | 0.857 | 1.000 | 1.000 | 0.667 | 1.000 | 1.000 | 1.000 | 0.182 | 0.000 |
| 5y85B | 1.000 | 1.000 | 0.952 | 0.900 | 0.952 | 0.947 | 0.857 | 0.909 | 1.000 | 0.900 | 0.909 | 0.952 |
| 6qn3A | 1.000 | 1.000 | 0.909 | 0.957 | 1.000 | 1.000 | 1.000 | 0.957 | 1.000 | 1.000 | 1.000 | 0.857 |
| 6ufjA | 0.952 | 0.952 | 1.000 | 0.957 | 0.818 | 0.900 | 0.900 | 0.900 | 0.900 | 0.952 | 0.909 | 0.952 |
| 6r47A | 1.000 | 1.000 | 0.909 | 0.909 | 0.909 | 0.952 | 0.900 | 0.900 | 0.952 | 1.000 | 1.000 | 1.000 |
| 2pxdB | 1.000 | 1.000 | 0.903 | 0.849 | 1.000 | 1.000 | 0.938 | 0.970 | 0.938 | 1.000 | 0.970 | 0.815 |
| 1u63B | 1.000 | 1.000 | 0.914 | 0.971 | 0.971 | 1.000 | 1.000 | 1.000 | 1.000 | 1.000 | 0.971 | 1.000 |
| 6fz0A | 0.960 | 0.960 | 0.692 | 0.786 | 0.960 | 0.960 | 0.960 | 0.833 | 0.957 | 0.923 | 0.880 | 1.000 |
| 4c7oE | 0.974 | 0.974 | 0.895 | 0.923 | 0.974 | 0.947 | 0.974 | 0.974 | 0.947 | 0.974 | 0.974 | 0.857 |
| 5dqkA | 0.909 | 0.909 | 0.833 | 0.833 | 0.909 | 0.909 | 0.909 | 0.833 | 0.909 | 0.833 | 0.769 | 0.909 |
| 5ztmC | 1.000 | 1.000 | 0.914 | 0.971 | 1.000 | 1.000 | 1.000 | 1.000 | 1.000 | 1.000 | 0.971 | 1.000 |
| 5k7dA | 0.957 | 0.957 | 0.889 | 0.923 | 0.857 | 0.909 | 0.909 | 0.909 | 0.857 | 0.957 | 0.957 | 0.909 |
| 1s03A | 1.000 | 1.000 | 0.971 | 0.973 | 0.973 | 0.973 | 0.973 | 1.000 | 1.000 | 1.000 | 0.944 | 1.000 |
| 4o26E | 1.000 | 1.000 | 0.849 | 0.909 | 1.000 | 0.970 | 0.909 | 0.867 | 0.938 | 0.970 | 0.867 | 0.938 |
| 4qjhB | 0.923 | 0.833 | 0.923 | 0.769 | 0.667 | 0.833 | 0.769 | 0.909 | 0.833 | 0.833 | 0.714 | 0.667 |
| 5xtmB | 0.889 | 0.889 | 0.800 | 0.800 | 0.778 | 0.778 | 0.824 | 0.706 | 0.824 | 0.778 | 0.778 | 0.824 |
| 1xjrA | 0.929 | 0.966 | 0.897 | 0.839 | 0.929 | 0.929 | 0.889 | 0.800 | 0.846 | 0.966 | 0.929 | 0.929 |
| 3iabR | 0.957 | 0.957 | 0.957 | 0.917 | 0.957 | 0.957 | 0.957 | 0.957 | 0.957 | 0.957 | 0.957 | 0.957 |
| 2nueC | 1.000 | 1.000 | 0.976 | 1.000 | 1.000 | 1.000 | 1.000 | 1.000 | 1.000 | 1.000 | 1.000 | 0.923 |
| 4qjdB | 1.000 | 1.000 | 1.000 | 0.857 | 0.923 | 0.923 | 0.923 | 0.833 | 0.857 | 0.923 | 0.933 | 1.000 |
| 6d3pA | 0.941 | 0.941 | 1.000 | 0.842 | 0.941 | 0.941 | 0.889 | 0.889 | 0.941 | 0.842 | 0.889 | 0.941 |
| 1p6vB | 1.000 | 1.000 | 1.000 | 0.960 | 0.917 | 1.000 | 0.960 | 0.960 | 1.000 | 1.000 | 0.923 | 1.000 |
| 4rmoB | 1.000 | 1.000 | 1.000 | 1.000 | 1.000 | 0.952 | 0.900 | 0.947 | 1.000 | 1.000 | 0.909 | 1.000 |
| 3zp8A | 0.889 | 0.889 | 0.750 | 0.889 | 0.889 | 0.727 | 0.727 | 0.800 | 0.889 | 0.889 | 0.727 | 0.889 |
| 5m0hA | 1.000 | 1.000 | 0.933 | 0.968 | 0.966 | 0.929 | 0.966 | 0.966 | 0.966 | 0.966 | 0.903 | 0.846 |
| 5nwqA | 0.957 | 0.957 | 0.870 | 0.870 | 0.957 | 0.909 | 0.909 | 0.909 | 0.957 | 0.957 | 0.957 | 0.957 |
| 4pmiA | 1.000 | 1.000 | 0.880 | 1.000 | 0.960 | 0.960 | 0.870 | 0.870 | 0.917 | 0.960 | 1.000 | 0.963 |

| | | | | | | | | | | | | |
|---|---|---|---|---|---|---|---|---|---|---|---|---|
| 5kk5B | 1.000 | 1.000 | 0.889 | 0.800 | 1.000 | 1.000 | 1.000 | 1.000 | 1.000 | 1.000 | 0.909 | 1.000 |
| 3p22A | 1.000 | 1.000 | 1.000 | 0.957 | 1.000 | 0.952 | 0.952 | 0.952 | 0.952 | 0.952 | 0.952 | 1.000 |
| 4kr6C | 0.957 | 0.957 | 0.818 | 0.917 | 0.957 | 0.957 | 0.957 | 0.909 | 0.909 | 0.917 | 0.957 | 0.857 |
| 6d12C | 0.960 | 0.960 | 0.880 | 0.880 | 0.960 | 0.923 | 0.960 | 0.960 | 0.960 | 0.917 | 0.923 | 0.917 |
| 6e8sA | 1.000 | 1.000 | 0.933 | 0.941 | 0.889 | 1.000 | 0.875 | 0.941 | 1.000 | 1.000 | 1.000 | 1.000 |
| 1zhoB | 1.000 | 1.000 | 1.000 | 1.000 | 1.000 | 1.000 | 1.000 | 0.963 | 1.000 | 1.000 | 1.000 | 0.963 |
| 4pdbI | 0.966 | 0.966 | 0.857 | 0.966 | 0.929 | 0.966 | 0.966 | 0.929 | 0.933 | 1.000 | 0.897 | 0.929 |
| 1f1tA | 1.000 | 0.960 | 0.917 | 0.833 | 1.000 | 0.880 | 0.800 | 0.917 | 0.917 | 0.917 | 1.000 | 0.762 |
| 1ylsB | 1.000 | 1.000 | 1.000 | 1.000 | 1.000 | 1.000 | 0.941 | 0.941 | 1.000 | 1.000 | 0.824 | 0.941 |
| 6dtdC | 0.909 | 0.909 | 0.909 | 0.957 | 0.909 | 0.818 | 0.857 | 0.800 | 0.800 | 0.818 | 0.909 | 0.857 |
| 1i6uC | 1.000 | 1.000 | 1.000 | 1.000 | 1.000 | 1.000 | 0.933 | 1.000 | 1.000 | 1.000 | 0.933 | 0.968 |
| 6sy4C | 1.000 | 1.000 | 0.933 | 0.824 | 0.933 | 0.933 | 0.800 | 1.000 | 0.857 | 1.000 | 0.933 | 0.923 |
| 1kogI | 0.960 | 0.960 | 0.960 | 0.923 | 0.960 | 0.960 | 0.960 | 0.960 | 0.960 | 0.960 | 1.000 | 0.917 |
| 5bjoE | 0.769 | 0.769 | 0.500 | 0.714 | 0.714 | 0.769 | 0.714 | 0.714 | 0.714 | 0.769 | 0.800 | 0.769 |
| 3gs5C | 0.857 | 0.952 | 0.870 | 0.870 | 0.957 | 0.909 | 0.952 | 0.952 | 0.909 | 0.952 | 0.917 | 0.818 |
| 6c65A | 1.000 | 1.000 | 0.909 | 1.000 | 1.000 | 1.000 | 1.000 | 0.909 | 0.909 | 0.909 | 1.000 | 1.000 |
| 4x4pB | 1.000 | 1.000 | 1.000 | 0.870 | 0.960 | 1.000 | 0.957 | 0.957 | 1.000 | 1.000 | 0.960 | 1.000 |
| 3ovbD | 1.000 | 1.000 | 0.957 | 1.000 | 1.000 | 0.960 | 1.000 | 0.960 | 1.000 | 1.000 | 1.000 | 1.000 |
| 6dcbB | 1.000 | 1.000 | 0.941 | 0.941 | 1.000 | 1.000 | 1.000 | 1.000 | 1.000 | 1.000 | 1.000 | 0.947 |
| 1et4A | 0.857 | 0.857 | 0.667 | 0.588 | 0.857 | 0.857 | 0.667 | 0.667 | 0.769 | 0.857 | 0.769 | 0.769 |
| 2xdbG | 0.933 | 0.933 | 0.875 | 0.875 | 0.933 | 0.875 | 0.933 | 1.000 | 0.933 | 0.933 | 0.875 | 1.000 |
| 5deaA | 1.000 | 0.923 | 0.667 | 0.714 | 0.769 | 0.923 | 0.667 | 0.857 | 1.000 | 0.923 | 0.769 | 0.923 |
| 6cf2G | 1.000 | 1.000 | 0.909 | 0.923 | 1.000 | 1.000 | 0.957 | 0.957 | 0.800 | 0.909 | 0.957 | 0.909 |
| 4v83AV | 0.966 | 0.929 | 0.929 | 0.966 | 0.929 | 0.889 | 0.889 | 0.889 | 0.889 | 0.889 | 0.966 | 0.889 |
| 2zh3B | 1.000 | 1.000 | 1.000 | 1.000 | 1.000 | 1.000 | 1.000 | 1.000 | 1.000 | 0.960 | 0.917 | 1.000 |
| 4oogD | 1.000 | 1.000 | 1.000 | 1.000 | 1.000 | 1.000 | 1.000 | 1.000 | 1.000 | 1.000 | 1.000 | 1.000 |
| 4atoG | 1.000 | 1.000 | 0.933 | 0.933 | 1.000 | 1.000 | 0.923 | 0.923 | 1.000 | 1.000 | 0.857 | 0.923 |
| 3gcaA | 0.923 | 0.923 | 0.769 | 0.833 | 0.833 | 0.923 | 0.667 | 0.727 | 0.833 | 0.923 | 0.833 | 0.833 |
| 3fu2B | 1.000 | 1.000 | 0.875 | 1.000 | 0.933 | 0.933 | 1.000 | 0.933 | 1.000 | 1.000 | 1.000 | 1.000 |
| 5v3fA | 1.000 | 1.000 | 0.857 | 1.000 | 1.000 | 1.000 | 1.000 | 1.000 | 1.000 | 0.889 | 1.000 | 1.000 |
| 6h0rA | 1.000 | 1.000 | 0.960 | 0.923 | 0.960 | 0.960 | 0.960 | 0.960 | 1.000 | 1.000 | 1.000 | 1.000 |
| 5voeA | 1.000 | 1.000 | 0.923 | 0.923 | 0.909 | 0.909 | 0.909 | 0.909 | 0.909 | 0.909 | 1.000 | 0.909 |
| 1jbrD | 0.933 | 0.933 | 0.857 | 0.933 | 0.933 | 0.933 | 0.875 | 0.933 | 0.933 | 0.933 | 0.941 | 0.933 |
| 2b63R | 1.000 | 0.941 | 1.000 | 1.000 | 0.941 | 0.941 | 0.941 | 0.941 | 0.941 | 0.941 | 0.941 | 1.000 |
| 3snpC | 0.957 | 0.909 | 0.909 | 0.909 | 0.957 | 0.957 | 0.909 | 0.909 | 0.909 | 0.909 | 0.870 | 0.909 |
| 5y58X | 1.000 | 0.947 | 0.842 | 0.947 | 0.947 | 0.947 | 0.947 | 1.000 | 0.947 | 0.947 | 0.947 | 1.000 |
| Average | 0.948 | 0.944 | 0.863 | 0.881 | 0.912 | 0.919 | 0.900 | 0.891 | 0.916 | 0.921 | 0.892 | 0.891 |

**Table S2** Target-specific F1-score of RNAstructure, RNAView, MC-Annotate and that of all atom types supported by pdb2ss (P, O5', C5', C4', C3' and O3') for 361 benchmark RNAs.

| RNA | RNAstructure | RNAView | MC-Annotate | P | O5' | C5' | C4' | C3' | O3' |
|---|---|---|---|---|---|---|---|---|---|
| 5g2xA | 0.480 | 0.696 | 0.741 | 0.047 | 0.327 | 0.437 | 0.451 | 0.420 | 0.340 |
| 6c4hA | 0.258 | 0.980 | 0.946 | 0.291 | 0.466 | 0.663 | 0.710 | 0.657 | 0.554 |
| 6hiwCA | 0.113 | 0.949 | 0.863 | 0.192 | 0.301 | 0.455 | 0.498 | 0.462 | 0.346 |
| 6chrA | 0.830 | 0.930 | 0.896 | 0.349 | 0.485 | 0.670 | 0.748 | 0.631 | 0.568 |
| 6hixAA | 0.000 | 0.977 | 0.894 | 0.193 | 0.338 | 0.546 | 0.588 | 0.487 | 0.394 |
| 6n7rR | 0.823 | 0.981 | 0.937 | 0.240 | 0.275 | 0.789 | 0.870 | 0.771 | 0.614 |
| 1fg0A | 0.610 | 0.972 | 0.943 | 0.438 | 0.584 | 0.718 | 0.772 | 0.723 | 0.643 |
| 5wlcL0 | 0.775 | 0.926 | 0.938 | 0.488 | 0.636 | 0.792 | 0.893 | 0.837 | 0.710 |
| 5zwnP | 0.551 | 0.931 | 0.929 | 0.421 | 0.547 | 0.761 | 0.776 | 0.701 | 0.561 |
| 6swe2 | 0.465 | 0.957 | 0.947 | 0.339 | 0.513 | 0.680 | 0.745 | 0.692 | 0.547 |
| 5j01A | 0.740 | 0.993 | 0.989 | 0.397 | 0.558 | 0.758 | 0.802 | 0.708 | 0.614 |
| 6agbA | 0.603 | 0.878 | 0.924 | 0.392 | 0.510 | 0.753 | 0.702 | 0.657 | 0.597 |
| 6q9a4 | 0.403 | 0.851 | 0.757 | 0.260 | 0.349 | 0.599 | 0.540 | 0.432 | 0.304 |
| 3bwpA | 0.495 | 0.987 | 0.974 | 0.426 | 0.569 | 0.774 | 0.802 | 0.720 | 0.595 |
| 3q1qB | 0.581 | 0.986 | 0.982 | 0.399 | 0.547 | 0.790 | 0.853 | 0.769 | 0.623 |
| 6ahuA | 0.641 | 0.907 | 0.932 | 0.384 | 0.515 | 0.735 | 0.794 | 0.703 | 0.593 |
| 6w2s0 | 0.352 | 0.835 | 0.835 | 0.294 | 0.364 | 0.488 | 0.512 | 0.550 | 0.415 |
| 6q954 | 0.239 | 0.883 | 0.879 | 0.309 | 0.421 | 0.614 | 0.717 | 0.480 | 0.452 |
| 5u4ja | 0.525 | 0.982 | 0.949 | 0.361 | 0.571 | 0.735 | 0.856 | 0.667 | 0.580 |
| 2a64A | 0.541 | 0.957 | 0.990 | 0.000 | 0.643 | 0.802 | 0.851 | 0.768 | 0.677 |
| 6g901 | 0.135 | 0.916 | 0.917 | 0.428 | 0.539 | 0.754 | 0.817 | 0.672 | 0.632 |
| 6sxoL5 | 0.663 | 0.962 | 0.975 | 0.386 | 0.488 | 0.712 | 0.781 | 0.634 | 0.607 |
| 5flxz | 0.519 | 0.987 | 0.956 | 0.465 | 0.622 | 0.800 | 0.854 | 0.802 | 0.679 |
| 6frk1 | 0.709 | 0.955 | 0.938 | 0.518 | 0.610 | 0.767 | 0.819 | 0.725 | 0.714 |
| 5a8lA | 0.000 | 1.000 | 0.868 | 0.277 | 0.504 | 0.471 | 0.561 | 0.562 | 0.500 |
| 1x8wB | 0.676 | 0.971 | 0.973 | 0.013 | 0.540 | 0.737 | 0.838 | 0.732 | 0.724 |
| 5tc1R | 0.309 | 0.659 | 0.784 | 0.525 | 0.605 | 0.553 | 0.720 | 0.628 | 0.582 |
| 5oql2 | 0.436 | 0.930 | 0.918 | 0.441 | 0.541 | 0.757 | 0.848 | 0.736 | 0.645 |
| 1y0qA | 0.810 | 0.946 | 0.927 | 0.029 | 0.587 | 0.833 | 0.861 | 0.743 | 0.703 |
| 6j6qL | 0.622 | 0.959 | 0.959 | 0.455 | 0.547 | 0.758 | 0.881 | 0.706 | 0.640 |
| 6p5n1 | 0.655 | 0.800 | 0.901 | 0.379 | 0.462 | 0.623 | 0.661 | 0.632 | 0.555 |
| 5zwmH | 0.714 | 0.919 | 0.928 | 0.421 | 0.570 | 0.817 | 0.704 | 0.586 | 0.587 |
| 5jupEC | 0.687 | 0.971 | 0.981 | 0.410 | 0.544 | 0.758 | 0.782 | 0.599 | 0.565 |
| 1u6bB | 0.832 | 0.991 | 0.965 | 0.500 | 0.646 | 0.800 | 0.881 | 0.717 | 0.693 |
| 6d904 | 0.520 | 0.894 | 0.807 | 0.294 | 0.412 | 0.526 | 0.625 | 0.530 | 0.472 |
| 4gmaZ | 0.339 | 0.917 | 0.946 | 0.426 | 0.522 | 0.596 | 0.806 | 0.715 | 0.611 |
| 6gyvA | 0.505 | 0.992 | 0.952 | 0.016 | 0.659 | 0.789 | 0.909 | 0.851 | 0.659 |
| 5v3iA | 0.793 | 0.974 | 0.927 | 0.588 | 0.694 | 0.868 | 0.916 | 0.759 | 0.769 |
| 3jcs3 | 0.391 | 0.921 | 0.837 | 0.323 | 0.566 | 0.646 | 0.750 | 0.646 | 0.511 |
| 6az34 | 0.878 | 0.958 | 0.950 | 0.497 | 0.573 | 0.783 | 0.922 | 0.736 | 0.686 |
| 6j6gD | 0.866 | 0.947 | 0.979 | 0.000 | 0.720 | 0.882 | 0.849 | 0.769 | 0.756 |
| 3dilA | 0.763 | 1.000 | 0.954 | 0.438 | 0.574 | 0.803 | 0.837 | 0.631 | 0.611 |
| 5lj3Z | 0.713 | 0.989 | 0.989 | 0.560 | 0.710 | 0.936 | 0.936 | 0.838 | 0.746 |
| 5t2aE | 0.467 | 0.941 | 0.933 | 0.467 | 0.577 | 0.819 | 0.857 | 0.785 | 0.603 |

| | | | | | | | | | |
|---|---|---|---|---|---|---|---|---|---|
| 3p49A | 0.833 | 0.907 | 0.816 | 0.398 | 0.605 | 0.750 | 0.826 | 0.734 | 0.605 |
| 5wlcL2 | 0.294 | 0.933 | 0.875 | 0.314 | 0.500 | 0.712 | 0.712 | 0.689 | 0.550 |
| 6ufhA | 0.667 | 0.978 | 0.957 | 0.494 | 0.591 | 0.871 | 0.871 | 0.662 | 0.642 |
| 6qx91 | 0.899 | 1.000 | 0.941 | 0.388 | 0.507 | 0.726 | 0.857 | 0.784 | 0.667 |
| 6az37 | 0.167 | 1.000 | 0.862 | 0.000 | 0.421 | 0.648 | 0.868 | 0.754 | 0.667 |
| 6ft62 | 0.375 | 0.979 | 0.868 | 0.474 | 0.468 | 0.721 | 0.746 | 0.579 | 0.564 |
| 3pdrA | 0.898 | 0.990 | 0.970 | 0.453 | 0.612 | 0.764 | 0.817 | 0.716 | 0.639 |
| 4gxyA | 0.723 | 0.958 | 0.942 | 0.443 | 0.584 | 0.189 | 0.220 | 0.226 | 0.269 |
| 6oleD | 0.281 | 0.952 | 0.933 | 0.388 | 0.563 | 0.816 | 0.784 | 0.702 | 0.580 |
| 5xxb4 | 0.174 | 0.930 | 0.826 | 0.400 | 0.507 | 0.692 | 0.655 | 0.731 | 0.585 |
| 1u9sA | 0.787 | 0.980 | 0.970 | 0.511 | 0.653 | 0.807 | 0.951 | 0.817 | 0.733 |
| 5xy34 | 0.271 | 0.889 | 0.906 | 0.479 | 0.587 | 0.688 | 0.800 | 0.772 | 0.750 |
| 4v8pB2 | 0.161 | 1.000 | 0.952 | 0.396 | 0.460 | 0.750 | 0.837 | 0.735 | 0.632 |
| 3j79C | 0.320 | 1.000 | 0.912 | 0.495 | 0.633 | 0.812 | 0.881 | 0.853 | 0.776 |
| 1nbsB | 0.767 | 0.988 | 0.977 | 0.503 | 0.667 | 0.882 | 0.911 | 0.804 | 0.695 |
| 6jdvB | 0.977 | 1.000 | 0.968 | 0.533 | 0.615 | 0.815 | 0.889 | 0.786 | 0.698 |
| 6id0H | 0.516 | 0.933 | 0.889 | 0.432 | 0.667 | 0.865 | 0.727 | 0.653 | 0.653 |
| 3hhnC | 0.765 | 0.989 | 0.978 | 0.000 | 0.723 | 0.811 | 0.915 | 0.843 | 0.718 |
| 3ndbM | 0.903 | 0.966 | 0.936 | 0.011 | 0.651 | 0.772 | 0.705 | 0.583 | 0.562 |
| 6exn2 | 0.743 | 1.000 | 0.985 | 0.529 | 0.667 | 0.927 | 0.901 | 0.821 | 0.727 |
| 6ahdI | 0.710 | 1.000 | 0.982 | 0.593 | 0.620 | 0.915 | 0.947 | 0.900 | 0.761 |
| 3l3cS | 0.000 | 0.952 | 0.906 | 0.419 | 0.491 | 0.674 | 0.716 | 0.660 | 0.492 |
| 4uykR | 0.837 | 1.000 | 0.947 | 0.419 | 0.579 | 0.776 | 0.874 | 0.763 | 0.682 |
| 2il9A | 0.197 | 0.750 | 0.649 | 0.000 | 0.323 | 0.600 | 0.577 | 0.416 | 0.395 |
| 6n5qA | 0.592 | 1.000 | 0.923 | 0.368 | 0.520 | 0.725 | 0.868 | 0.611 | 0.647 |
| 3v7eC | 0.932 | 0.978 | 0.968 | 0.000 | 0.705 | 0.871 | 0.905 | 0.775 | 0.733 |
| 5ganV | 0.267 | 0.882 | 0.974 | 0.487 | 0.655 | 0.723 | 0.923 | 0.694 | 0.597 |
| 2z75B | 0.762 | 1.000 | 1.000 | 0.525 | 0.667 | 0.000 | 0.000 | 0.000 | 0.646 |
| 5zluX | 0.667 | 0.892 | 0.925 | 0.441 | 0.577 | 0.763 | 0.806 | 0.895 | 0.621 |
| 1vq89 | 0.886 | 1.000 | 0.944 | 0.000 | 0.680 | 0.857 | 0.805 | 0.767 | 0.719 |
| 5dm6Y | 0.754 | 0.986 | 0.914 | 0.531 | 0.648 | 0.907 | 0.907 | 0.931 | 0.809 |
| 6t4qC4 | 0.831 | 1.000 | 0.937 | 0.576 | 0.687 | 0.864 | 0.843 | 0.875 | 0.783 |
| 5mmiB | 0.817 | 0.987 | 0.961 | 0.541 | 0.686 | 0.800 | 0.847 | 0.809 | 0.667 |
| 4ybbDB | 0.270 | 1.000 | 0.960 | 0.534 | 0.660 | 0.805 | 0.875 | 0.824 | 0.714 |
| 6ek0L7 | 0.857 | 0.986 | 0.947 | 0.000 | 0.707 | 0.795 | 0.854 | 0.778 | 0.795 |
| 1nbsA | 0.552 | 0.984 | 0.968 | 0.588 | 0.747 | 0.886 | 0.899 | 0.775 | 0.674 |
| 6qdv2 | 0.627 | 0.971 | 0.944 | 0.632 | 0.681 | 0.900 | 1.000 | 0.872 | 0.766 |
| 4v8pB3 | 0.778 | 0.986 | 0.973 | 0.511 | 0.747 | 0.875 | 0.875 | 0.722 | 0.700 |
| 3jcs8 | 0.333 | 0.979 | 0.778 | 0.455 | 0.568 | 0.733 | 0.746 | 0.597 | 0.590 |
| 4kqyA | 0.763 | 0.974 | 0.923 | 0.015 | 0.712 | 0.907 | 0.839 | 0.804 | 0.750 |
| 6uesA | 0.300 | 0.952 | 0.966 | 0.537 | 0.612 | 0.752 | 0.812 | 0.796 | 0.707 |
| 5xxb3 | 0.880 | 0.928 | 0.972 | 0.500 | 0.620 | 0.750 | 0.872 | 0.778 | 0.695 |
| 3j79B | 0.327 | 0.986 | 0.973 | 0.538 | 0.680 | 0.786 | 0.843 | 0.814 | 0.824 |
| 5o60B | 0.909 | 0.969 | 0.971 | 0.561 | 0.681 | 0.775 | 0.927 | 0.901 | 0.800 |
| 6rm3L70 | 0.833 | 0.941 | 0.901 | 0.571 | 0.645 | 0.846 | 0.831 | 0.805 | 0.773 |
| 6xyw3 | 0.467 | 0.960 | 0.852 | 0.000 | 0.505 | 0.724 | 0.840 | 0.648 | 0.667 |
| 4qlnA | 0.361 | 1.000 | 0.974 | 0.398 | 0.518 | 0.758 | 0.923 | 0.774 | 0.713 |
| 5xy33 | 0.000 | 0.929 | 0.897 | 0.403 | 0.549 | 0.754 | 0.839 | 0.725 | 0.651 |

| | | | | | | | | | |
|---|---|---|---|---|---|---|---|---|---|
| 4qk9A | 0.254 | 1.000 | 0.950 | 0.380 | 0.567 | 0.758 | 0.774 | 0.692 | 0.654 |
| 6s0xB | 0.500 | 1.000 | 0.949 | 0.000 | 0.602 | 0.761 | 0.761 | 0.750 | 0.649 |
| 5wtiB | 0.557 | 1.000 | 0.968 | 0.462 | 0.682 | 0.811 | 0.829 | 0.699 | 0.667 |
| 6v3aB | 0.636 | 0.966 | 0.921 | 0.491 | 0.659 | 0.712 | 0.871 | 0.691 | 0.683 |
| 5f9rA | 0.754 | 0.964 | 0.912 | 0.487 | 0.658 | 0.862 | 0.875 | 0.806 | 0.757 |
| 6ahdB | 0.862 | 0.966 | 0.951 | 0.455 | 0.515 | 0.750 | 0.931 | 0.818 | 0.607 |
| 5t5hD | 0.000 | 0.985 | 0.877 | 0.522 | 0.632 | 0.698 | 0.909 | 0.734 | 0.706 |
| 6ha1B | 0.657 | 0.984 | 0.925 | 0.000 | 0.622 | 0.882 | 0.845 | 0.800 | 0.638 |
| 5vt0R | 0.841 | 0.984 | 0.912 | 0.554 | 0.619 | 0.800 | 0.892 | 0.824 | 0.732 |
| 6ol3C | 0.966 | 1.000 | 0.989 | 0.536 | 0.634 | 0.885 | 0.911 | 0.829 | 0.829 |
| 3jb9P | 0.444 | 1.000 | 1.000 | 0.271 | 0.311 | 0.593 | 0.667 | 0.667 | 0.762 |
| 6ny2B | 0.780 | 0.933 | 0.969 | 0.571 | 0.698 | 0.785 | 0.892 | 0.734 | 0.675 |
| 6ck5A | 0.824 | 0.986 | 0.919 | 0.194 | 0.261 | 0.427 | 0.795 | 0.782 | 0.703 |
| 3f2qX | 0.632 | 1.000 | 0.969 | 0.481 | 0.627 | 0.747 | 0.861 | 0.810 | 0.703 |
| 4y1mB | 0.861 | 1.000 | 0.973 | 0.493 | 0.595 | 0.791 | 0.900 | 0.783 | 0.654 |
| 6dlrA | 0.787 | 1.000 | 0.955 | 0.457 | 0.531 | 0.870 | 0.870 | 0.800 | 0.714 |
| 6eriAB | 0.851 | 1.000 | 0.980 | 0.485 | 0.657 | 0.739 | 0.923 | 0.762 | 0.800 |
| 4wflA | 0.358 | 1.000 | 0.972 | 0.424 | 0.567 | 0.814 | 0.875 | 0.814 | 0.737 |
| 3jb9C | 0.915 | 0.983 | 0.984 | 0.558 | 0.644 | 0.839 | 0.931 | 0.794 | 0.711 |
| 6j6gE | 0.727 | 0.970 | 1.000 | 0.485 | 0.711 | 0.865 | 0.970 | 0.970 | 0.744 |
| 4lckC | 0.842 | 0.947 | 0.912 | 0.542 | 0.707 | 0.915 | 0.831 | 0.711 | 0.750 |
| 4frnA | 0.622 | 0.949 | 0.968 | 0.474 | 0.643 | 0.754 | 0.912 | 0.818 | 0.692 |
| 2xxaF | 0.400 | 0.971 | 0.986 | 0.574 | 0.700 | 0.833 | 0.854 | 0.713 | 0.786 |
| 6mj0A | 0.419 | 0.983 | 0.951 | 0.538 | 0.635 | 0.767 | 0.836 | 0.778 | 0.709 |
| 4w90C | 0.612 | 0.929 | 0.871 | 0.015 | 0.551 | 0.740 | 0.806 | 0.750 | 0.667 |
| 5u33B | 0.490 | 0.982 | 0.929 | 0.551 | 0.617 | 0.750 | 0.915 | 0.776 | 0.853 |
| 6dmcA | 0.848 | 1.000 | 0.939 | 0.530 | 0.645 | 0.912 | 0.873 | 0.816 | 0.827 |
| 4y1jA | 0.831 | 1.000 | 1.000 | 0.455 | 0.653 | 0.733 | 0.868 | 0.710 | 0.695 |
| 3suhX | 0.656 | 0.983 | 0.936 | 0.452 | 0.583 | 0.853 | 0.829 | 0.817 | 0.611 |
| 4rzdA | 0.820 | 0.982 | 0.966 | 0.134 | 0.622 | 0.849 | 0.849 | 0.849 | 0.727 |
| 6n2vA | 0.969 | 1.000 | 0.985 | 0.504 | 0.711 | 0.853 | 0.821 | 0.821 | 0.744 |
| 6az35 | 0.263 | 0.958 | 0.941 | 0.444 | 0.686 | 0.828 | 0.873 | 0.774 | 0.739 |
| 6id1F | 0.774 | 1.000 | 0.963 | 0.545 | 0.774 | 0.960 | 0.960 | 0.857 | 0.800 |
| 4oquA | 0.712 | 1.000 | 0.970 | 0.566 | 0.719 | 0.865 | 0.927 | 0.877 | 0.736 |
| 6jxmB | 0.947 | 0.947 | 0.951 | 0.538 | 0.609 | 0.765 | 0.771 | 0.767 | 0.747 |
| 6jooB | 0.643 | 1.000 | 0.981 | 0.549 | 0.608 | 0.794 | 0.862 | 0.781 | 0.714 |
| 3ktwC | 0.667 | 0.926 | 0.853 | 0.029 | 0.611 | 0.730 | 0.879 | 0.720 | 0.562 |
| 6dvkH | 0.939 | 0.984 | 0.968 | 0.559 | 0.689 | 0.849 | 0.954 | 0.833 | 0.729 |
| 2yghA | 0.893 | 0.983 | 0.951 | 0.557 | 0.635 | 0.794 | 0.740 | 0.761 | 0.789 |
| 5ml7A | 0.814 | 0.983 | 0.947 | 0.596 | 0.596 | 0.849 | 0.778 | 0.761 | 0.757 |
| 5b2oB | 0.625 | 1.000 | 0.933 | 0.494 | 0.679 | 0.449 | 0.537 | 0.471 | 0.714 |
| 5x2hB | 0.732 | 1.000 | 0.960 | 0.480 | 0.667 | 0.923 | 0.889 | 0.960 | 0.750 |
| 3iwnA | 0.815 | 1.000 | 0.983 | 0.021 | 0.651 | 0.794 | 0.871 | 0.831 | 0.781 |
| 1m5oB | 0.864 | 0.974 | 0.952 | 0.000 | 0.667 | 0.816 | 0.833 | 0.704 | 0.576 |
| 4rumA | 0.862 | 1.000 | 0.967 | 0.691 | 0.767 | 0.849 | 0.889 | 0.767 | 0.767 |
| 3culD | 0.966 | 0.983 | 0.933 | 0.552 | 0.586 | 0.800 | 0.918 | 0.889 | 0.707 |
| 6mwnA | 0.806 | 0.983 | 0.906 | 0.476 | 0.594 | 0.845 | 0.844 | 0.727 | 0.683 |
| 5t83A | 0.210 | 1.000 | 0.939 | 0.500 | 0.556 | 0.035 | 0.033 | 0.028 | 0.033 |

| | | | | | | | | | |
|---|---|---|---|---|---|---|---|---|---|
| 4lvwA | 0.702 | 0.983 | 0.952 | 0.435 | 0.514 | 0.759 | 0.909 | 0.732 | 0.659 |
| 5osg2 | 0.098 | 0.828 | 0.788 | 0.000 | 0.640 | 0.780 | 0.732 | 0.711 | 0.525 |
| 1wz2C | 0.526 | 0.958 | 0.980 | 0.326 | 0.442 | 0.719 | 0.793 | 0.639 | 0.535 |
| 3owiB | 0.941 | 1.000 | 1.000 | 0.650 | 0.694 | 0.781 | 0.889 | 0.926 | 0.781 |
| 4v8bAB | 0.542 | 0.878 | 0.884 | 0.412 | 0.526 | 0.731 | 0.800 | 0.655 | 0.583 |
| 3k0jE | 0.522 | 0.978 | 0.920 | 0.000 | 0.730 | 0.836 | 0.807 | 0.821 | 0.807 |
| 6c0f6 | 0.744 | 0.971 | 0.947 | 0.395 | 0.615 | 0.809 | 0.895 | 0.773 | 0.586 |
| 5aoxF | 0.877 | 1.000 | 0.984 | 0.446 | 0.607 | 0.411 | 0.484 | 0.421 | 0.636 |
| 3q1qC | 0.846 | 0.980 | 1.000 | 0.435 | 0.558 | 0.794 | 0.746 | 0.806 | 0.735 |
| 4v8dAB | 0.913 | 0.872 | 0.850 | 0.462 | 0.575 | 0.737 | 0.714 | 0.508 | 0.613 |
| 4mgnA | 0.342 | 0.978 | 0.978 | 0.560 | 0.609 | 0.894 | 0.913 | 0.750 | 0.645 |
| 5u3gB | 0.833 | 0.958 | 0.906 | 0.500 | 0.553 | 0.786 | 0.772 | 0.746 | 0.721 |
| 6b14R | 0.696 | 1.000 | 0.943 | 0.469 | 0.556 | 0.755 | 0.746 | 0.702 | 0.635 |
| 4yazA | 0.682 | 1.000 | 1.000 | 0.516 | 0.716 | 0.828 | 0.960 | 0.906 | 0.814 |
| 6jq5A | 0.829 | 1.000 | 0.950 | 0.000 | 0.643 | 0.818 | 0.800 | 0.679 | 0.571 |
| 3w3sB | 0.349 | 0.985 | 0.985 | 0.388 | 0.464 | 0.736 | 0.914 | 0.727 | 0.598 |
| 3am1B | 0.848 | 0.983 | 1.000 | 0.433 | 0.592 | 0.763 | 0.817 | 0.763 | 0.598 |
| 5lzdy | 0.386 | 1.000 | 1.000 | 0.466 | 0.582 | 0.759 | 0.846 | 0.795 | 0.710 |
| 3umyB | 0.684 | 1.000 | 0.978 | 0.350 | 0.447 | 0.778 | 0.913 | 0.712 | 0.597 |
| 6ah3T | 0.400 | 0.976 | 0.952 | 0.449 | 0.603 | 0.870 | 0.870 | 0.800 | 0.615 |
| 3adbC | 0.000 | 1.000 | 1.000 | 0.460 | 0.556 | 0.842 | 0.821 | 0.762 | 0.688 |
| 6cu1A | 0.800 | 1.000 | 0.983 | 0.540 | 0.600 | 0.831 | 0.844 | 0.750 | 0.711 |
| 5on2B | 0.231 | 0.939 | 0.981 | 0.439 | 0.714 | 0.758 | 0.848 | 0.781 | 0.694 |
| 3amtB | 0.723 | 0.900 | 0.955 | 0.512 | 0.636 | 0.741 | 0.800 | 0.727 | 0.585 |
| 6rjaD | 0.514 | 0.950 | 0.976 | 0.482 | 0.571 | 0.833 | 0.976 | 0.769 | 0.702 |
| 4v8nAW | 0.154 | 0.737 | 0.692 | 0.000 | 0.340 | 0.593 | 0.457 | 0.474 | 0.489 |
| 6v5bD | 0.943 | 0.982 | 0.915 | 0.529 | 0.614 | 0.839 | 0.963 | 0.754 | 0.658 |
| 5vppQV | 0.821 | 0.839 | 0.941 | 0.360 | 0.486 | 0.640 | 0.549 | 0.557 | 0.607 |
| 6uggA | 0.652 | 1.000 | 0.978 | 0.452 | 0.526 | 0.737 | 0.857 | 0.712 | 0.609 |
| 3d2vA | 0.632 | 1.000 | 0.976 | 0.372 | 0.493 | 0.792 | 0.884 | 0.760 | 0.691 |
| 5ccbN | 0.652 | 0.978 | 0.958 | 0.500 | 0.656 | 0.724 | 0.778 | 0.840 | 0.646 |
| 6svsA | 0.864 | 1.000 | 1.000 | 0.361 | 0.511 | 0.800 | 0.814 | 0.686 | 0.605 |
| 5j8bx | 0.976 | 0.976 | 0.977 | 0.417 | 0.548 | 0.833 | 0.816 | 0.816 | 0.714 |
| 2zzmB | 0.000 | 0.857 | 0.952 | 0.379 | 0.471 | 0.632 | 0.792 | 0.717 | 0.537 |
| 6ufmA | 0.952 | 1.000 | 0.978 | 0.429 | 0.583 | 0.894 | 0.778 | 0.646 | 0.600 |
| 6v3av | 0.842 | 1.000 | 0.930 | 0.029 | 0.833 | 1.000 | 0.833 | 0.800 | 0.741 |
| 1h3eB | 0.000 | 1.000 | 1.000 | 0.485 | 0.632 | 0.787 | 0.828 | 0.774 | 0.649 |
| 6i0yV | 0.927 | 0.950 | 0.923 | 0.444 | 0.533 | 0.696 | 0.588 | 0.679 | 0.582 |
| 1j2bC | 0.778 | 1.000 | 0.974 | 0.463 | 0.633 | 0.808 | 0.974 | 0.927 | 0.643 |
| 5o2rx | 0.304 | 0.976 | 0.952 | 0.518 | 0.677 | 0.875 | 0.955 | 0.875 | 0.677 |
| 3a2kC | 0.478 | 1.000 | 1.000 | 0.525 | 0.690 | 0.769 | 0.857 | 0.764 | 0.689 |
| 6q977 | 0.857 | 0.947 | 0.950 | 0.361 | 0.597 | 0.425 | 0.857 | 0.878 | 0.653 |
| 5ah5D | 0.160 | 1.000 | 0.939 | 0.500 | 0.688 | 0.772 | 0.898 | 0.772 | 0.733 |
| 4jf2A | 0.884 | 1.000 | 0.960 | 0.552 | 0.727 | 0.873 | 0.889 | 0.762 | 0.828 |
| 6t4q6 | 0.486 | 1.000 | 0.955 | 0.449 | 0.506 | 0.755 | 0.755 | 0.755 | 0.597 |
| 6az12 | 0.789 | 0.786 | 0.938 | 0.000 | 0.378 | 0.556 | 0.833 | 0.578 | 0.509 |
| 1qf6B | 0.698 | 1.000 | 0.955 | 0.556 | 0.656 | 0.800 | 0.784 | 0.714 | 0.833 |
| 6r5q2 | 0.621 | 0.800 | 0.824 | 0.000 | 0.528 | 0.757 | 0.683 | 0.609 | 0.528 |

| | | | | | | | | | |
|---|---|---|---|---|---|---|---|---|---|
| 2iy5T | 0.977 | 0.927 | 1.000 | 0.053 | 0.654 | 0.756 | 0.720 | 0.870 | 0.481 |
| 4v5lAY | 0.977 | 0.977 | 0.930 | 0.423 | 0.553 | 0.824 | 0.824 | 0.746 | 0.556 |
| 5el63K | 0.485 | 0.750 | 0.833 | 0.000 | 0.324 | 0.431 | 0.564 | 0.512 | 0.478 |
| 6t7t6 | 0.714 | 1.000 | 1.000 | 0.433 | 0.571 | 0.824 | 0.894 | 0.894 | 0.737 |
| 4wj4B | 0.500 | 1.000 | 0.936 | 0.462 | 0.636 | 0.857 | 0.894 | 0.808 | 0.737 |
| 5lzsii | 0.529 | 0.971 | 0.895 | 0.000 | 0.479 | 0.766 | 0.692 | 0.735 | 0.590 |
| 6r87B | 0.270 | 0.950 | 0.950 | 0.358 | 0.464 | 0.630 | 0.809 | 0.618 | 0.471 |
| 4v5gAY | 0.667 | 0.783 | 0.889 | 0.317 | 0.414 | 0.688 | 0.611 | 0.542 | 0.415 |
| 6q9a7 | 0.545 | 0.929 | 0.867 | 0.316 | 0.381 | 0.615 | 0.706 | 0.585 | 0.387 |
| 2zueB | 0.651 | 0.977 | 0.978 | 0.447 | 0.600 | 0.677 | 0.840 | 0.764 | 0.792 |
| 1g59B | 0.727 | 1.000 | 0.936 | 0.452 | 0.525 | 0.909 | 0.857 | 0.750 | 0.609 |
| 4rdxC | 0.000 | 0.976 | 1.000 | 0.526 | 0.691 | 0.816 | 0.833 | 0.702 | 0.714 |
| 5x6bP | 0.884 | 0.947 | 0.950 | 0.396 | 0.507 | 0.691 | 0.857 | 0.720 | 0.581 |
| 1il2C | 0.368 | 0.955 | 0.955 | 0.421 | 0.500 | 0.870 | 0.870 | 0.714 | 0.656 |
| 6t4q7 | 0.800 | 1.000 | 1.000 | 0.479 | 0.567 | 0.791 | 0.708 | 0.739 | 0.576 |
| 2csxC | 0.976 | 0.976 | 0.952 | 0.469 | 0.506 | 0.800 | 0.851 | 0.667 | 0.690 |
| 6tbvPTR | 0.977 | 1.000 | 0.955 | 0.560 | 0.778 | 0.778 | 0.824 | 0.840 | 0.808 |
| 6tb3n | 0.977 | 1.000 | 0.978 | 0.538 | 0.609 | 0.826 | 0.824 | 0.792 | 0.627 |
| 6qdwv | 0.400 | 0.800 | 0.429 | 0.119 | 0.130 | 0.174 | 0.167 | 0.500 | 0.286 |
| 1gaxC | 0.615 | 0.952 | 0.930 | 0.519 | 0.625 | 0.737 | 0.764 | 0.724 | 0.764 |
| 6ek0S6 | 0.540 | 0.636 | 0.714 | 0.028 | 0.449 | 0.667 | 0.690 | 0.529 | 0.513 |
| 2dr2B | 0.950 | 0.973 | 0.950 | 0.520 | 0.667 | 1.000 | 0.760 | 0.679 | 0.603 |
| 4v7lAY | 0.727 | 0.963 | 0.857 | 0.000 | 0.433 | 0.605 | 0.533 | 0.578 | 0.481 |
| 4lckB | 0.578 | 1.000 | 0.978 | 0.447 | 0.635 | 0.792 | 0.857 | 0.792 | 0.840 |
| 3wqyC | 0.304 | 1.000 | 0.958 | 0.543 | 0.595 | 0.759 | 0.880 | 0.830 | 0.759 |
| 4v9iAY | 0.000 | 0.833 | 0.588 | 0.000 | 0.308 | 0.500 | 0.364 | 0.444 | 0.308 |
| 6ip5zu | 0.429 | 0.952 | 0.930 | 0.000 | 0.486 | 0.745 | 0.717 | 0.679 | 0.691 |
| 1j1uB | 0.977 | 1.000 | 0.978 | 0.000 | 0.576 | 0.778 | 0.778 | 0.764 | 0.724 |
| 3rg5A | 0.000 | 1.000 | 0.968 | 0.475 | 0.602 | 0.862 | 0.778 | 0.667 | 0.737 |
| 1u0bA | 0.711 | 1.000 | 0.952 | 0.444 | 0.613 | 0.889 | 0.816 | 0.784 | 0.635 |
| 4yyeC | 0.595 | 1.000 | 0.955 | 0.392 | 0.540 | 0.714 | 0.851 | 0.816 | 0.513 |
| 4prfB | 0.000 | 1.000 | 0.978 | 0.500 | 0.764 | 0.772 | 0.898 | 0.880 | 0.786 |
| 3q3zA | 0.545 | 1.000 | 0.960 | 0.495 | 0.730 | 0.754 | 0.821 | 0.807 | 0.767 |
| 5d8hA | 0.718 | 1.000 | 0.895 | 0.471 | 0.653 | 0.800 | 0.762 | 0.696 | 0.582 |
| 3akzE | 0.977 | 0.952 | 0.977 | 0.408 | 0.494 | 0.689 | 0.824 | 0.609 | 0.585 |
| 2d6fF | 0.900 | 0.977 | 0.936 | 0.442 | 0.519 | 0.778 | 0.833 | 0.714 | 0.635 |
| 3j7a7 | 0.000 | 0.750 | 0.600 | 0.037 | 0.235 | 0.518 | 0.387 | 0.293 | 0.378 |
| 2dlcY | 0.865 | 0.970 | 0.971 | 0.395 | 0.542 | 0.000 | 0.125 | 0.000 | 0.082 |
| 5czzB | 0.722 | 1.000 | 0.970 | 0.508 | 0.638 | 0.865 | 0.941 | 0.889 | 0.711 |
| 6cae1y | 0.286 | 0.923 | 0.952 | 0.374 | 0.500 | 0.800 | 0.857 | 0.706 | 0.545 |
| 5e6mC | 0.778 | 0.977 | 0.933 | 0.412 | 0.519 | 0.800 | 0.755 | 0.741 | 0.606 |
| 4znpA | 0.833 | 1.000 | 0.952 | 0.556 | 0.632 | 0.816 | 0.816 | 0.851 | 0.755 |
| 2derC | 0.000 | 0.875 | 1.000 | 0.430 | 0.500 | 0.651 | 0.714 | 0.566 | 0.582 |
| 5wt3C | 0.744 | 1.000 | 0.979 | 0.389 | 0.500 | 0.750 | 0.840 | 0.746 | 0.579 |
| 5ud5D | 0.979 | 1.000 | 0.980 | 0.469 | 0.575 | 0.708 | 0.958 | 0.868 | 0.719 |
| 5wwrC | 0.529 | 0.952 | 1.000 | 0.460 | 0.576 | 0.741 | 0.690 | 0.784 | 0.702 |
| 2zniC | 0.927 | 0.927 | 0.927 | 0.396 | 0.556 | 0.731 | 0.851 | 0.741 | 0.645 |
| 6lxdD | 0.756 | 0.884 | 0.875 | 0.458 | 0.609 | 0.786 | 0.737 | 0.724 | 0.647 |

| | | | | | | | | | |
|---|---|---|---|---|---|---|---|---|---|
| 3la5A | 0.889 | 0.980 | 0.923 | 0.506 | 0.579 | 0.759 | 0.898 | 0.772 | 0.588 |
| 5kpyA | 0.800 | 1.000 | 1.000 | 0.436 | 0.571 | 0.923 | 0.900 | 0.818 | 0.654 |
| 6az36 | 0.000 | 0.947 | 0.952 | 0.518 | 0.286 | 0.667 | 0.778 | 0.857 | 0.762 |
| 2du3D | 0.533 | 0.947 | 0.950 | 0.388 | 0.613 | 0.775 | 0.927 | 0.792 | 0.623 |
| 2oiuP | 0.930 | 1.000 | 1.000 | 0.541 | 0.830 | 0.978 | 0.898 | 0.936 | 0.863 |
| 5tpyA | 0.640 | 1.000 | 1.000 | 0.476 | 0.686 | 0.787 | 0.941 | 0.842 | 0.686 |
| 6gazAV | 0.545 | 1.000 | 0.947 | 0.000 | 0.588 | 0.872 | 0.971 | 0.829 | 0.829 |
| 1kxkA | 0.808 | 1.000 | 1.000 | 0.000 | 0.623 | 0.846 | 0.939 | 0.868 | 0.742 |
| 5ob3A | 0.465 | 1.000 | 0.955 | 0.425 | 0.581 | 0.800 | 0.826 | 0.755 | 0.667 |
| 3ivnA | 0.884 | 0.979 | 0.960 | 0.000 | 0.571 | 0.746 | 0.917 | 0.764 | 0.603 |
| 3ephE | 0.650 | 0.895 | 0.900 | 0.370 | 0.607 | 0.654 | 0.607 | 0.708 | 0.548 |
| 6u8dA | 0.962 | 1.000 | 1.000 | 0.565 | 0.732 | 0.812 | 0.946 | 0.900 | 0.750 |
| 2qusA | 0.952 | 1.000 | 0.978 | 0.560 | 0.714 | 0.833 | 0.870 | 0.870 | 0.755 |
| 4pqvA | 0.312 | 0.976 | 1.000 | 0.538 | 0.727 | 0.837 | 0.976 | 0.930 | 0.816 |
| 6p2hA | 0.667 | 1.000 | 1.000 | 0.432 | 0.644 | 0.905 | 0.905 | 0.760 | 0.576 |
| 4wzjV | 0.818 | 0.952 | 0.955 | 0.000 | 0.667 | 0.889 | 0.930 | 0.933 | 0.678 |
| 4fe5B | 0.878 | 0.978 | 0.917 | 0.048 | 0.636 | 0.678 | 0.870 | 0.741 | 0.640 |
| 3skiB | 0.889 | 0.980 | 0.980 | 0.489 | 0.697 | 0.852 | 0.807 | 0.793 | 0.697 |
| 6gawBB | 0.973 | 0.971 | 0.923 | 0.590 | 0.727 | 0.821 | 0.900 | 0.947 | 0.818 |
| 5a8lB | 0.000 | 0.900 | 0.870 | 0.457 | 0.552 | 0.750 | 0.727 | 0.857 | 0.643 |
| 1h4sT | 0.000 | 1.000 | 0.976 | 0.469 | 0.655 | 0.864 | 0.905 | 0.844 | 0.775 |
| 3r4fA | 1.000 | 0.978 | 0.958 | 0.000 | 0.676 | 0.780 | 0.920 | 0.836 | 0.767 |
| 3egzB | 0.970 | 0.971 | 0.919 | 0.607 | 0.723 | 0.882 | 0.850 | 0.773 | 0.895 |
| 4xwfA | 0.722 | 1.000 | 1.000 | 0.513 | 0.706 | 0.905 | 0.950 | 0.950 | 0.808 |
| 3nkbB | 0.778 | 1.000 | 0.974 | 0.543 | 0.643 | 0.844 | 0.950 | 0.905 | 0.760 |
| 5b63D | 0.909 | 0.875 | 1.000 | 0.416 | 0.456 | 0.667 | 0.636 | 0.941 | 0.593 |
| 6rflU | 0.849 | 1.000 | 0.947 | 0.479 | 0.667 | 0.829 | 0.919 | 0.773 | 0.791 |
| 5t5aA | 0.256 | 0.979 | 0.979 | 0.000 | 0.667 | 0.846 | 0.957 | 0.880 | 0.721 |
| 5btpA | 0.333 | 1.000 | 0.976 | 0.067 | 0.679 | 0.745 | 0.844 | 0.884 | 0.633 |
| 2czjB | 0.800 | 0.941 | 1.000 | 0.415 | 0.557 | 0.667 | 0.769 | 0.708 | 0.567 |
| 3icqD | 0.333 | 0.897 | 0.897 | 0.394 | 0.605 | 0.718 | 0.700 | 0.700 | 0.549 |
| 2hvyE | 0.889 | 1.000 | 0.970 | 0.508 | 0.000 | 0.000 | 0.000 | 0.100 | 0.000 |
| 5ddpA | 0.970 | 1.000 | 1.000 | 0.000 | 0.821 | 0.914 | 0.970 | 0.970 | 0.711 |
| 1un6E | 0.839 | 0.941 | 0.944 | 0.000 | 0.818 | 0.923 | 0.923 | 0.878 | 0.735 |
| 6db8R | 0.857 | 1.000 | 0.950 | 0.576 | 0.654 | 0.723 | 0.895 | 0.773 | 0.809 |
| 3rw6F | 1.000 | 1.000 | 0.974 | 0.551 | 0.704 | 0.884 | 0.864 | 0.760 | 0.633 |
| 6pmoA | 0.950 | 0.950 | 0.952 | 0.148 | 0.585 | 0.792 | 0.905 | 0.826 | 0.760 |
| 4m4oB | 0.833 | 1.000 | 0.930 | 0.434 | 0.597 | 0.829 | 0.818 | 0.694 | 0.667 |
| 3lwrD | 0.941 | 1.000 | 1.000 | 0.000 | 0.627 | 0.842 | 0.727 | 0.604 | 0.627 |
| 4v2sQ | 0.667 | 0.971 | 0.971 | 0.000 | 0.000 | 0.046 | 0.054 | 0.000 | 0.136 |
| 1dk1B | 0.857 | 1.000 | 0.974 | 0.000 | 0.667 | 0.900 | 0.900 | 0.818 | 0.735 |
| 3j7yB | 0.690 | 1.000 | 0.919 | 0.643 | 0.750 | 0.971 | 1.000 | 1.000 | 0.783 |
| 4rgeA | 0.125 | 1.000 | 1.000 | 0.000 | 0.597 | 0.717 | 0.974 | 0.889 | 0.136 |
| 1serT | 0.000 | 1.000 | 1.000 | 0.336 | 0.469 | 0.760 | 0.844 | 0.111 | 0.258 |
| 2nreF | 0.370 | 0.750 | 0.857 | 0.390 | 0.564 | 0.700 | 0.757 | 0.686 | 0.619 |
| 5lyuA | 0.000 | 1.000 | 1.000 | 0.538 | 0.714 | 0.913 | 0.857 | 0.894 | 0.933 |
| 4k27U | 1.000 | 1.000 | 0.850 | 0.486 | 0.708 | 0.895 | 1.000 | 0.756 | 0.694 |
| 1mzpB | 0.385 | 1.000 | 0.966 | 0.394 | 0.491 | 0.867 | 0.867 | 0.750 | 0.578 |

| | | | | | | | | | |
|---|---|---|---|---|---|---|---|---|---|
| 6laxA | 0.970 | 1.000 | 0.970 | 0.500 | 0.615 | 0.821 | 0.889 | 0.889 | 0.762 |
| 5uq8x | 0.778 | 0.923 | 0.857 | 0.000 | 0.609 | 0.933 | 0.778 | 1.000 | 0.625 |
| 4pkdV | 0.968 | 1.000 | 0.968 | 0.462 | 0.625 | 0.750 | 0.789 | 0.732 | 0.789 |
| 2fk6R | 0.938 | 0.970 | 0.971 | 0.066 | 0.696 | 0.865 | 0.744 | 0.889 | 0.682 |
| 3e5cA | 0.769 | 1.000 | 0.970 | 0.571 | 0.744 | 0.842 | 0.941 | 0.842 | 0.800 |
| 4encA | 0.370 | 1.000 | 1.000 | 0.508 | 0.789 | 0.909 | 0.938 | 0.938 | 0.682 |
| 4ojiA | 0.788 | 1.000 | 1.000 | 0.000 | 0.766 | 0.850 | 0.944 | 0.878 | 0.766 |
| 5tf6D | 0.000 | 0.900 | 0.900 | 0.500 | 0.786 | 0.720 | 0.900 | 0.583 | 0.560 |
| 6aayB | 0.870 | 1.000 | 0.952 | 0.500 | 0.588 | 0.952 | 1.000 | 0.909 | 0.909 |
| 2qwyA | 0.444 | 1.000 | 0.929 | 0.429 | 0.615 | 0.774 | 0.960 | 0.800 | 0.750 |
| 6iv8B | 0.889 | 1.000 | 0.941 | 0.762 | 0.667 | 0.889 | 0.889 | 0.800 | 0.842 |
| 3npqA | 0.429 | 0.880 | 0.929 | 0.585 | 0.727 | 0.727 | 0.800 | 0.923 | 0.727 |
| 5y7mB | 0.762 | 1.000 | 0.960 | 0.489 | 0.688 | 0.759 | 0.880 | 0.846 | 0.688 |
| 5xwyB | 0.889 | 1.000 | 0.769 | 0.381 | 0.889 | 0.889 | 0.889 | 0.889 | 0.889 |
| 6e9eB | 0.000 | 0.800 | 0.667 | 0.261 | 0.545 | 1.000 | 1.000 | 0.500 | 0.429 |
| 5y85B | 0.833 | 1.000 | 1.000 | 0.540 | 0.667 | 0.833 | 0.909 | 0.769 | 0.667 |
| 6qn3A | 0.833 | 0.952 | 0.917 | 0.000 | 0.579 | 1.000 | 0.917 | 0.846 | 0.688 |
| 6ufjA | 0.471 | 1.000 | 0.957 | 0.000 | 0.571 | 0.625 | 0.870 | 0.870 | 0.800 |
| 6r47A | 0.381 | 1.000 | 0.957 | 0.000 | 0.000 | 0.667 | 0.917 | 0.917 | 0.846 |
| 2pxdB | 1.000 | 1.000 | 0.970 | 0.000 | 0.667 | 0.780 | 0.941 | 0.889 | 0.681 |
| 1u63B | 0.941 | 0.970 | 1.000 | 0.500 | 0.694 | 0.791 | 0.971 | 0.872 | 0.739 |
| 6fz0A | 0.375 | 1.000 | 1.000 | 0.043 | 0.727 | 0.960 | 0.960 | 0.800 | 0.706 |
| 4c7oE | 0.895 | 0.974 | 1.000 | 0.528 | 0.667 | 0.844 | 0.927 | 0.844 | 0.844 |
| 5dqkA | 0.667 | 1.000 | 0.923 | 0.476 | 0.526 | 0.909 | 0.769 | 0.667 | 0.556 |
| 5ztmC | 0.919 | 1.000 | 0.944 | 0.523 | 0.708 | 0.944 | 1.000 | 1.000 | 0.694 |
| 5k7dA | 0.600 | 1.000 | 0.917 | 0.585 | 0.611 | 0.815 | 0.957 | 0.880 | 0.733 |
| 1s03A | 0.889 | 1.000 | 1.000 | 0.562 | 0.735 | 0.837 | 0.923 | 0.818 | 0.720 |
| 4o26E | 0.914 | 1.000 | 1.000 | 0.479 | 0.667 | 0.872 | 1.000 | 0.791 | 0.696 |
| 4qjhB | 0.571 | 0.909 | 1.000 | 0.706 | 0.833 | 0.625 | 0.833 | 0.714 | 0.750 |
| 5xtmB | 0.316 | 1.000 | 0.952 | 0.488 | 0.645 | 0.909 | 0.909 | 0.769 | 0.714 |
| 1xjrA | 0.929 | 1.000 | 0.968 | 0.667 | 0.800 | 0.903 | 0.903 | 0.849 | 0.828 |
| 3iabR | 1.000 | 1.000 | 1.000 | 0.686 | 0.815 | 1.000 | 0.889 | 0.923 | 0.800 |
| 2nueC | 1.000 | 1.000 | 1.000 | 0.000 | 0.700 | 0.824 | 0.933 | 0.857 | 0.656 |
| 4qjdB | 0.000 | 1.000 | 1.000 | 0.560 | 0.737 | 0.875 | 1.000 | 0.875 | 0.875 |
| 6d3pA | 0.941 | 1.000 | 1.000 | 0.593 | 0.615 | 0.727 | 0.941 | 0.800 | 0.667 |
| 1p6vB | 1.000 | 1.000 | 1.000 | 0.000 | 0.857 | 0.800 | 1.000 | 0.857 | 0.727 |
| 4rmoB | 0.000 | 1.000 | 0.952 | 0.049 | 0.769 | 1.000 | 1.000 | 0.690 | 0.588 |
| 3zp8A | 0.727 | 1.000 | 0.909 | 0.381 | 0.533 | 0.889 | 0.615 | 0.533 | 0.500 |
| 5m0hA | 1.000 | 0.889 | 0.966 | 0.000 | 0.698 | 0.788 | 0.903 | 1.000 | 0.788 |
| 5nwqA | 0.609 | 1.000 | 1.000 | 0.000 | 0.710 | 0.815 | 0.960 | 0.846 | 0.710 |
| 4pmiA | 0.870 | 1.000 | 0.929 | 0.491 | 0.650 | 1.000 | 1.000 | 0.929 | 0.897 |
| 5kk5B | 0.000 | 1.000 | 0.909 | 0.000 | 0.444 | 1.000 | 1.000 | 0.833 | 1.000 |
| 3p22A | 1.000 | 1.000 | 1.000 | 0.468 | 0.564 | 0.815 | 0.846 | 0.611 | 0.579 |
| 4kr6C | 0.917 | 0.909 | 0.957 | 0.000 | 0.750 | 0.889 | 0.846 | 0.733 | 0.846 |
| 6d12C | 0.923 | 0.923 | 0.923 | 0.558 | 0.585 | 0.774 | 0.857 | 0.857 | 0.774 |
| 6e8sA | 0.889 | 1.000 | 1.000 | 0.046 | 0.421 | 0.571 | 0.696 | 0.727 | 0.667 |
| 1zhoB | 1.000 | 1.000 | 1.000 | 0.441 | 0.615 | 0.722 | 1.000 | 0.929 | 0.743 |
| 4pdbI | 0.889 | 1.000 | 0.966 | 0.431 | 0.667 | 0.875 | 0.875 | 0.800 | 0.778 |

| | | | | | | | | | |
|---|---|---|---|---|---|---|---|---|---|
| 1f1tA | 1.000 | 1.000 | 1.000 | 0.000 | 0.929 | 1.000 | 0.929 | 0.929 | 0.929 |
| 1ylsB | 0.421 | 1.000 | 0.941 | 0.000 | 0.889 | 1.000 | 0.800 | 0.667 | 0.615 |
| 6dtdC | 0.870 | 0.957 | 0.923 | 0.550 | 0.815 | 0.714 | 0.857 | 0.720 | 0.643 |
| 1i6uC | 0.867 | 1.000 | 1.000 | 0.556 | 0.698 | 0.909 | 0.857 | 0.833 | 0.789 |
| 6sy4C | 1.000 | 0.923 | 0.875 | 0.000 | 0.778 | 0.000 | 0.000 | 0.000 | 0.000 |
| 1kogI | 1.000 | 1.000 | 1.000 | 0.650 | 0.703 | 0.929 | 0.929 | 0.867 | 0.743 |
| 5bjoE | 0.625 | 0.933 | 1.000 | 0.000 | 0.632 | 0.667 | 0.769 | 0.632 | 0.500 |
| 3gs5C | 0.696 | 0.952 | 0.917 | 0.077 | 0.769 | 0.870 | 0.952 | 0.800 | 0.741 |
| 6c65A | 1.000 | 1.000 | 1.000 | 0.000 | 0.750 | 0.857 | 1.000 | 1.000 | 0.857 |
| 4x4pB | 1.000 | 1.000 | 0.957 | 0.750 | 0.686 | 0.828 | 0.857 | 0.857 | 0.815 |
| 3ovbD | 1.000 | 0.957 | 0.957 | 0.000 | 0.564 | 0.857 | 0.923 | 0.889 | 0.774 |
| 6dcbB | 0.737 | 1.000 | 1.000 | 0.750 | 0.900 | 0.857 | 1.000 | 1.000 | 0.818 |
| 1et4A | 0.133 | 1.000 | 0.889 | 0.414 | 0.556 | 0.625 | 0.769 | 0.769 | 0.526 |
| 2xdbG | 0.667 | 1.000 | 0.933 | 0.000 | 0.636 | 0.933 | 0.824 | 0.824 | 0.667 |
| 5deaA | 0.667 | 1.000 | 1.000 | 0.000 | 0.476 | 0.875 | 0.778 | 0.778 | 0.824 |
| 6cf2G | 1.000 | 0.957 | 0.917 | 0.857 | 0.923 | 0.923 | 0.923 | 1.000 | 0.889 |
| 4v83AV | 0.692 | 0.966 | 1.000 | 0.000 | 0.698 | 0.849 | 0.737 | 0.667 | 0.688 |
| 2zh3B | 1.000 | 1.000 | 1.000 | 0.468 | 0.606 | 0.857 | 1.000 | 0.889 | 0.649 |
| 4oogD | 1.000 | 1.000 | 1.000 | 0.596 | 0.757 | 0.933 | 1.000 | 1.000 | 0.849 |
| 4atoG | 0.000 | 1.000 | 0.933 | 0.000 | 0.636 | 1.000 | 1.000 | 1.000 | 0.667 |
| 3gcaA | 0.833 | 1.000 | 1.000 | 0.000 | 0.833 | 0.833 | 0.714 | 0.833 | 0.625 |
| 3fu2B | 0.714 | 1.000 | 0.941 | 0.000 | 0.889 | 0.000 | 0.000 | 0.000 | 0.167 |
| 5v3fA | 0.000 | 1.000 | 1.000 | 0.000 | 0.800 | 1.000 | 1.000 | 1.000 | 1.000 |
| 6h0rA | 1.000 | 1.000 | 1.000 | 0.160 | 0.531 | 0.634 | 0.812 | 0.565 | 0.765 |
| 5voeA | 0.500 | 1.000 | 0.923 | 0.000 | 0.833 | 0.833 | 0.833 | 0.769 | 0.625 |
| 1jbrD | 0.933 | 1.000 | 1.000 | 0.000 | 0.824 | 0.933 | 0.933 | 0.737 | 0.667 |
| 2b63R | 1.000 | 1.000 | 1.000 | 0.000 | 1.000 | 1.000 | 1.000 | 0.900 | 0.750 |
| 3snpC | 0.957 | 1.000 | 1.000 | 0.524 | 0.556 | 0.000 | 0.000 | 0.000 | 0.846 |
| 5y58X | 1.000 | 1.000 | 1.000 | 0.667 | 0.909 | 0.769 | 0.870 | 0.870 | 0.692 |
| Average | 0.644 | 0.965 | 0.942 | 0.362 | 0.607 | 0.768 | 0.816 | 0.744 | 0.662 |

**Table S3** F1-score of different base pair types for all atom types supported by CSSR for 361 benchmark RNAs.

† "All" means using all supported atoms. CSSR supported any combination of its 10 types of atoms. Due to limitation of space, we only show two combinations: all atoms, and C1'C4'P atoms.

‡ "N" is atom N9 and N1 for purine and pyrimidine, respectively.

| Base pair type | All† | C1'C4'P | P | O5' | C5' | C4' | C3' | C2' | C1' | O4' | O3' | N‡ |
|---|---|---|---|---|---|---|---|---|---|---|---|---|
| Watson-Crick (A:U) | 0.925 | 0.919 | 0.832 | 0.854 | 0.884 | 0.889 | 0.874 | 0.857 | 0.883 | 0.892 | 0.848 | 0.875 |
| Watson-Crick (G:C) | 0.956 | 0.953 | 0.887 | 0.907 | 0.930 | 0.935 | 0.919 | 0.913 | 0.929 | 0.936 | 0.920 | 0.897 |
| Wobble (G:U) | 0.664 | 0.662 | 0.562 | 0.569 | 0.612 | 0.620 | 0.586 | 0.584 | 0.619 | 0.620 | 0.588 | 0.613 |

**Table S4** Target-specific F1-score of all atom types supported by CSSR for 21 RNA-puzzle targets.

# The data for RNA-puzzle problems 7, 16, 22, 23 are not yet publicly available. Problem 14 correspond to 2 RNA structures, one with ligand bound and the other in apo form.

† "All" means using all supported atoms. CSSR supported any combination of its 10 types of atoms. Due to limitation of space, we only show two combinations: all atoms, and C1'C4'P atoms.

‡ "N" is atom N9 and N1 for purine and pyrimidine, respectively.

| RNA-puzzle problem # | All | C1'C4'P | P | O5' | C5' | C4' | C3' | C2' | C1' | O4' | O3' | N' |
|---|---|---|---|---|---|---|---|---|---|---|---|---|
| 1. Regulatory element from human thymidylate synthase mRNA | 0.919 | 0.947 | 0.872 | 0.895 | 0.947 | 0.947 | 0.895 | 0.947 | 0.947 | 0.947 | 0.923 | 0.857 |
| 2. Self-assembling RNA square | 0.931 | 0.946 | 0.424 | 0.765 | 0.873 | 0.960 | 0.944 | 0.944 | 0.973 | 0.960 | 0.946 | 0.836 |
| 3. Glycine riboswitch | 0.917 | 0.917 | 0.894 | 0.894 | 0.917 | 0.917 | 0.894 | 0.894 | 0.894 | 0.917 | 0.898 | 0.917 |
| 4. SAM-I riboswitch aptamer | 0.956 | 0.944 | 0.923 | 0.956 | 0.933 | 0.923 | 0.921 | 0.935 | 0.923 | 0.956 | 0.956 | 0.966 |
| 5. Lariat capping ribozyme. | 0.928 | 0.935 | 0.803 | 0.903 | 0.912 | 0.919 | 0.885 | 0.869 | 0.911 | 0.919 | 0.874 | 0.879 |
| 6. Adenosylcobalamin riboswitch | 0.895 | 0.895 | 0.857 | 0.885 | 0.835 | 0.885 | 0.885 | 0.874 | 0.885 | 0.887 | 0.876 | 0.891 |
| 8. SAM riboswitch | 0.969 | 0.969 | 0.984 | 0.951 | 0.935 | 0.952 | 0.918 | 0.900 | 0.951 | 0.952 | 0.984 | 0.984 |
| 9. 5-hydroxytryptophan aptamer | 0.947 | 0.947 | 0.769 | 0.850 | 0.837 | 0.947 | 0.872 | 0.889 | 0.923 | 0.923 | 0.857 | 0.947 |
| 10. T-box riboswitch and tRNA | 0.968 | 0.968 | 0.875 | 0.866 | 0.925 | 0.923 | 0.878 | 0.913 | 0.945 | 0.957 | 0.938 | 0.957 |
| 11. 5'-terminal hairpin of the 7SK snRNA | 0.878 | 0.878 | 0.780 | 0.750 | 0.905 | 0.850 | 0.750 | 0.800 | 0.829 | 0.857 | 0.632 | 0.629 |
| 12. YdaO riboswitch | 0.935 | 0.909 | 0.872 | 0.886 | 0.895 | 0.907 | 0.935 | 0.919 | 0.919 | 0.904 | 0.921 | 0.866 |
| 13. ZMP riboswitch | 0.950 | 0.950 | 0.878 | 0.923 | 0.923 | 0.923 | 0.923 | 0.923 | 0.923 | 0.923 | 0.950 | 0.950 |
| 14. L-glutamine riboswitch (Bound) | 0.909 | 0.938 | 0.882 | 0.909 | 0.938 | 0.938 | 0.938 | 0.938 | 0.938 | 0.938 | 0.909 | 0.938 |
| 14. L-glutamine riboswitch (Free) | 1.000 | 1.000 | 0.944 | 0.941 | 0.970 | 0.970 | 0.941 | 0.970 | 0.970 | 0.970 | 1.000 | 1.000 |
| 15. Hammerhead | 0.800 | 0.821 | 0.769 | 0.821 | 0.821 | 0.821 | 0.800 | 0.780 | 0.821 | 0.800 | 0.750 | 0.769 |
| 17. Pistol ribozyme | 0.947 | 0.923 | 0.878 | 0.900 | 0.923 | 0.923 | 0.923 | 0.923 | 0.923 | 0.923 | 0.895 | 0.947 |
| 18. Exonuclease resistant RNA from Zika virus | 0.941 | 0.920 | 0.694 | 0.902 | 0.960 | 0.920 | 0.870 | 0.857 | 0.920 | 0.920 | 0.917 | 0.898 |
| 19. Twister sister | 0.933 | 0.933 | 0.773 | 0.936 | 0.889 | 0.889 | 0.682 | 0.864 | 0.933 | 0.936 | 0.476 | 0.933 |
| 20. Twister sister | 0.936 | 0.958 | 0.894 | 0.875 | 0.870 | 0.936 | 0.936 | 0.913 | 0.917 | 0.894 | 0.917 | 0.939 |
| 21. Guanidine III riboswitch | 0.870 | 0.870 | 0.957 | 0.957 | 0.870 | 0.870 | 0.909 | 0.909 | 0.870 | 0.870 | 0.870 | 0.909 |
| 24. Adenovirus Virus-Associated (VA) RNA I apical and central domains | 0.909 | 0.919 | 0.944 | 0.911 | 0.919 | 0.909 | 0.919 | 0.919 | 0.919 | 0.932 | 0.932 | 0.943 |
| Average | 0.926 | 0.928 | 0.841 | 0.889 | 0.905 | 0.916 | 0.886 | 0.899 | 0.916 | 0.918 | 0.877 | 0.903 |

**Table S5** Target-specific F1-score of RNAstructure, DSSR, ClaRNA, RNAView, MC-annotate and that of all atom types supported by pdb2ss (P, O6’, C5’, C4’, C3’ and O3’) for 21 RNA-puzzle targets.

† Since the ground-truth secondary structure assignment is derived from experimental structures while the assessed assignment is derived from computational structure models, the “DSSR” column does not always achieve a perfect F1-score of 1.000 even though both the ground-truth and the assessed assignment are from the same secondary structure assignment program.

‡ The TM-score$_{RNA}$ and RMSD between the selected RNA-puzzle structure model and the experimental structure are shown in the last two columns.

| RNA-puzzle problem | RNAstructure | DSSR † | ClaRNA | RNAView | MC-annotate | P | O6’ | C5’ | C4’ | C3’ | O3’ | TM-score | RMSD‡ |
|---|---|---|---|---|---|---|---|---|---|---|---|---|---|
| 1. Regulatory element from human thymidylate synthase mRNA | 0.000 | 0.923 | 0.923 | 0.923 | 0.900 | 0.468 | 0.610 | 0.947 | 0.947 | 0.692 | 0.750 | 0.369 | 4.1 |
| 2. Self-assembling RNA square | 0.000 | 0.946 | 0.916 | 0.901 | 0.919 | 0.659 | 0.681 | 0.840 | 0.935 | 0.911 | 0.819 | 0.866 | 1.7 |
| 3. Glycine riboswitch | 0.939 | 0.917 | 0.916 | 0.917 | 0.846 | 0.581 | 0.754 | 0.939 | 0.786 | 0.667 | 0.620 | 0.336 | 18.3 |
| 4. SAM-I riboswitch aptamer | 0.932 | 0.989 | 0.989 | 0.989 | 0.968 | 0.581 | 0.688 | 0.796 | 0.782 | 0.811 | 0.765 | 0.768 | 3.4 |
| 5. Lariat capping ribozyme. | 0.538 | 0.927 | 0.918 | 0.918 | 0.889 | 0.436 | 0.618 | 0.704 | 0.767 | 0.753 | 0.651 | 0.532 | 9.1 |
| 6. Adenosylcobalamin riboswitch | 0.562 | 0.914 | 0.914 | 0.914 | 0.895 | 0.469 | 0.559 | 0.671 | 0.862 | 0.780 | 0.653 | 0.426 | 14.3 |
| 8. SAM riboswitch | 0.881 | 0.968 | 0.984 | 0.984 | 0.954 | 0.554 | 0.667 | 0.827 | 0.886 | 0.838 | 0.775 | 0.580 | 4.5 |
| 9. 5-hydroxytryptophan aptamer | 0.800 | 0.947 | 0.895 | 0.923 | 0.829 | 0.387 | 0.576 | 0.706 | 0.818 | 0.632 | 0.526 | 0.459 | 6.2 |
| 10. T-box riboswitch and tRNA | 0.747 | 0.947 | 0.948 | 0.968 | 0.939 | 0.443 | 0.652 | 0.759 | 0.882 | 0.711 | 0.610 | 0.608 | 10.3 |
| 11. 5'-terminal hairpin of the 7SK snRNA | 0.927 | 0.850 | 0.850 | 0.811 | 0.820 | 0.493 | 0.513 | 0.741 | 0.930 | 0.633 | 0.491 | 0.341 | 5.5 |
| 12. YdaO riboswitch | 0.361 | 0.883 | 0.909 | 0.883 | 0.886 | 0.432 | 0.496 | 0.783 | 0.857 | 0.758 | 0.649 | 0.335 | 12.8 |
| 13. ZMP riboswitch | 0.684 | 0.950 | 0.950 | 0.950 | 0.927 | 0.528 | 0.603 | 0.905 | 0.905 | 0.792 | 0.760 | 0.393 | 5.3 |
| 14. L-glutamine riboswitch (Bound) | 0.970 | 0.909 | 0.909 | 0.909 | 0.909 | 0.561 | 0.683 | 0.938 | 0.857 | 0.882 | 0.600 | 0.364 | 16.5 |
| 14. L-glutamine riboswitch (Free) | 1.000 | 1.000 | 1.000 | 1.000 | 0.944 | 0.567 | 0.850 | 0.944 | 0.850 | 0.944 | 0.654 | 0.370 | 7.2 |
| 15. Hammerhead | 0.769 | 0.850 | 0.850 | 0.850 | 0.829 | 0.438 | 0.525 | 0.744 | 0.821 | 0.627 | 0.653 | 0.898 | 6.3 |
| 17. Pistol ribozyme | 0.400 | 0.900 | 0.923 | 0.900 | 0.900 | 0.474 | 0.735 | 0.800 | 0.837 | 0.783 | 0.735 | 0.360 | 9.0 |
| 18. Exonuclease resistant RNA from Zika virus | 0.640 | 0.962 | 0.941 | 0.898 | 0.962 | 0.459 | 0.581 | 0.800 | 0.941 | 0.772 | 0.633 | 0.501 | 3.2 |
| 19. Twister sister | 0.256 | 0.978 | 0.956 | 0.956 | 0.978 | 0.473 | 0.579 | 0.783 | 0.875 | 0.688 | 0.361 | 0.423 | 5.5 |
| 20. Twister sister | 0.609 | 0.979 | 0.979 | 0.958 | 0.920 | 0.483 | 0.583 | 0.808 | 0.821 | 0.917 | 0.815 | 0.450 | 5.1 |
| 21. Guanidine III riboswitch | 0.609 | 0.956 | 0.956 | 0.956 | 0.846 | 0.629 | 0.647 | 0.880 | 0.714 | 0.769 | 0.880 | 0.319 | 3.5 |
| 24. Adenovirus Virus-Associated (VA) RNA I apical and central domains | 0.966 | 0.921 | 0.921 | 0.921 | 0.863 | 0.608 | 0.641 | 0.796 | 0.766 | 0.706 | 0.626 | 0.520 | 4.8 |
| Average | 0.647 | 0.934 | 0.931 | 0.925 | 0.901 | 0.511 | 0.631 | 0.815 | 0.850 | 0.765 | 0.668 | 0.487 | 7.4 |

**Table S6** Target-specific F1-score of CSSR, pdb2ss and RNAstructure for rSS assignment of low-resolution experimental RNA structures with only P atoms, where a high-resolution full-atomic experimental structures is also available for the same RNAs.

† The TM-score$_{RNA}$, RMSD and sequence identity between the low- and high-resolution RNA structures are shown in the last two columns. The ground-truth rSS assignment is from running DSSR on the high-resolution structures. The nucleotide-level correspondence between the low- and high-resolution structure is derived from the pairwise structure alignment by RNA-align.

| Low-resolution structure | High-resolution structure | CSSR | pdb2ss | RNAstructure | TM-score † | RMSD † | Sequence identity † |
|---|---|---|---|---|---|---|---|
| 4v48BA | 4v4vAA | 0.850 | 0.356 | 0.426 | 0.995 | 1.49 | 0.987 |
| 1jgqA | 2e5lA | 0.836 | 0.364 | 0.566 | 0.978 | 3.04 | 0.988 |
| 4adx9 | 1yhq9 | 0.909 | 0.467 | 0.451 | 0.999 | 0.15 | 1.000 |
| 1zc8Z | 2om7C | 0.918 | 0.509 | 0.912 | 0.807 | 1.58 | 0.948 |
| 1qzbB | 1ob5B | 0.850 | 0.469 | 0.238 | 0.821 | 1.39 | 0.961 |
| 1eg0O | 7chdI | 0.900 | 0.408 | 0.976 | 0.776 | 2.21 | 0.947 |
| 2agnC | 1p5oA | 0.884 | 0.538 | 0.511 | 0.708 | 2.15 | 1.000 |
| 1gsgT | 1qtqB | 0.950 | 0.437 | 0.974 | 0.931 | 0.59 | 0.973 |
| 1pn8D | 1jgqD | 0.950 | 0.514 | 0.889 | 0.919 | 0.00 | 0.919 |
| 1r2wC | 1mmsC | 0.786 | 0.449 | 0.733 | 0.797 | 1.13 | 1.000 |
| 3ep2B | 3j0dA | 0.889 | 0.643 | 0.818 | 0.911 | 0.56 | 0.940 |
| 2r1gB | 2noqB | 0.929 | 0.546 | 0.774 | 0.922 | 0.24 | 0.938 |
| 2agnA | 1f84A | 0.667 | 0.333 | 1.000 | 1.000 | 0.00 | 1.000 |
| 3ep2D | 3degI | 0.923 | 0.480 | 0.714 | 0.591 | 0.63 | 0.966 |
| 1qzcC | 2x7nA | 0.909 | 0.909 | 0.667 | 0.929 | 0.00 | 0.929 |
| 1eg0M | 5mrxA | 1.000 | 0.500 | 0.500 | 0.833 | 0.30 | 0.962 |
| Average | | 0.884 | 0.495 | 0.697 | 0.870 | 0.97 | 0.966 |

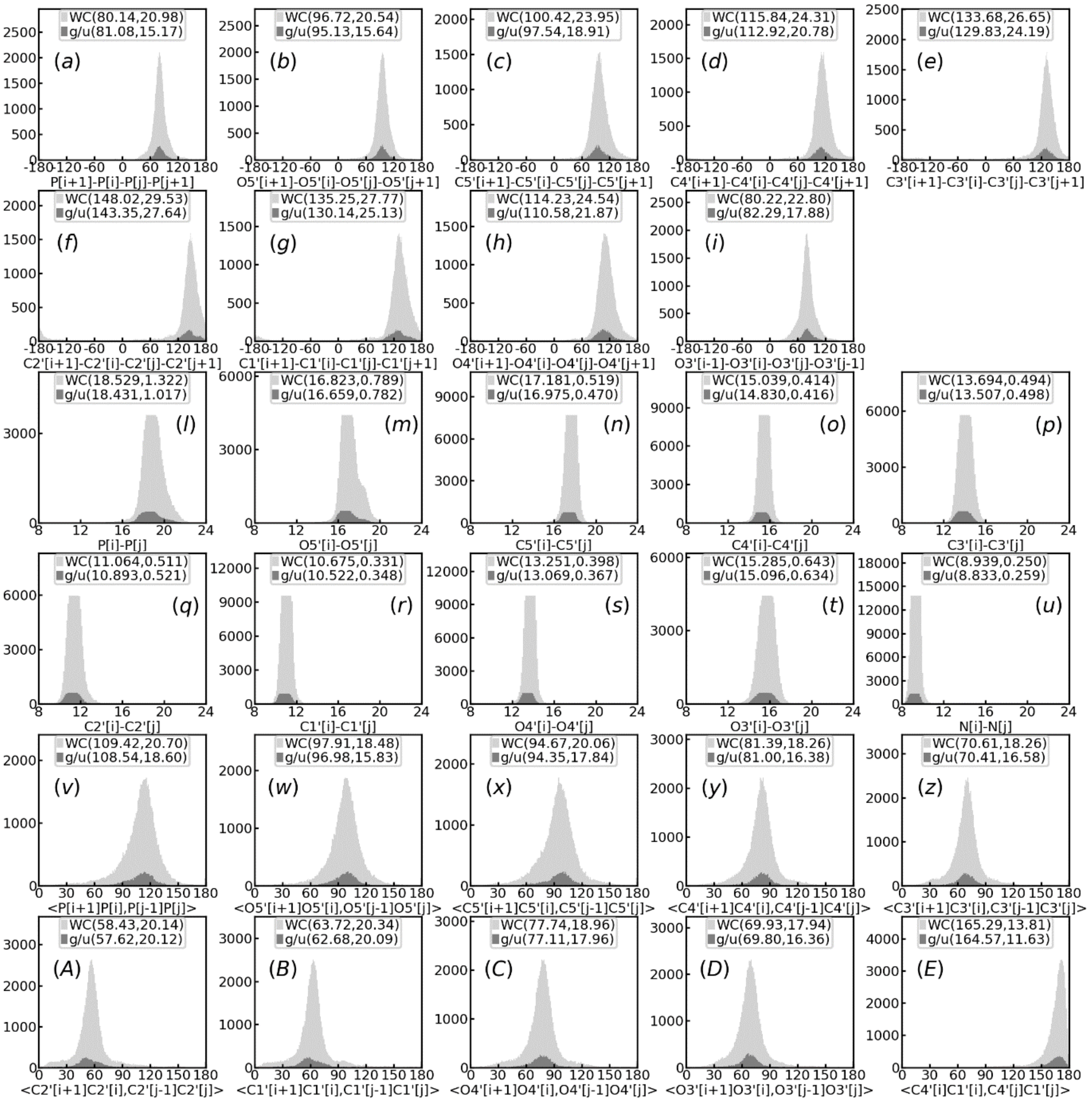


**Figure S1** Histogram for the distributions of dihedral angles ***(a-i)***, distances ***(l-u)*** and angles ***(v-E)*** formed by atoms across two nucleotides in canonical base pairs. Watson-Crick (WC) and G:U Wobble (g/u) base pairs are shown in light and dark grey, respectively. The statistics are obtained from 3724 non-redundant full atomic RNA chains. The mean and standard deviation for each distribution are listed within the parentheses. Since dihedral angles are periodic (e.g., a dihedral angle of 0 is the same as a dihedral angle of 360°), the mean and standard deviations are calculated twice, once in the range of 0 to 360° and another time in the range of -180° to 180°. Among these two sets of mean/standard deviation, the set with smaller standard deviation is chosen. Atoms used in the calculation are shown as x-axis labels. For example, "P[i]-P[j]" in ***(l)*** is the distance between P atoms of paired nucleotides *i* and *j* (*i*<*j*). "P[i+1]-P[i]-P[j]-P[j+1]" in ***(a)*** is the dihedral angle between the plane formed by P atoms from nucleotides *i*+1, *i* and *j*, and another plane formed by P atoms from

nucleotides *i*, *j* and *j*+1. “<P[i-1]P[i],P[j-1]P[j]>” in ***(v)*** is the angle between the vector pointing from P atoms of nucleotide *i*-1 to *i* and the vector pointing from P atoms of nucleotide *j*-1 to *j*.

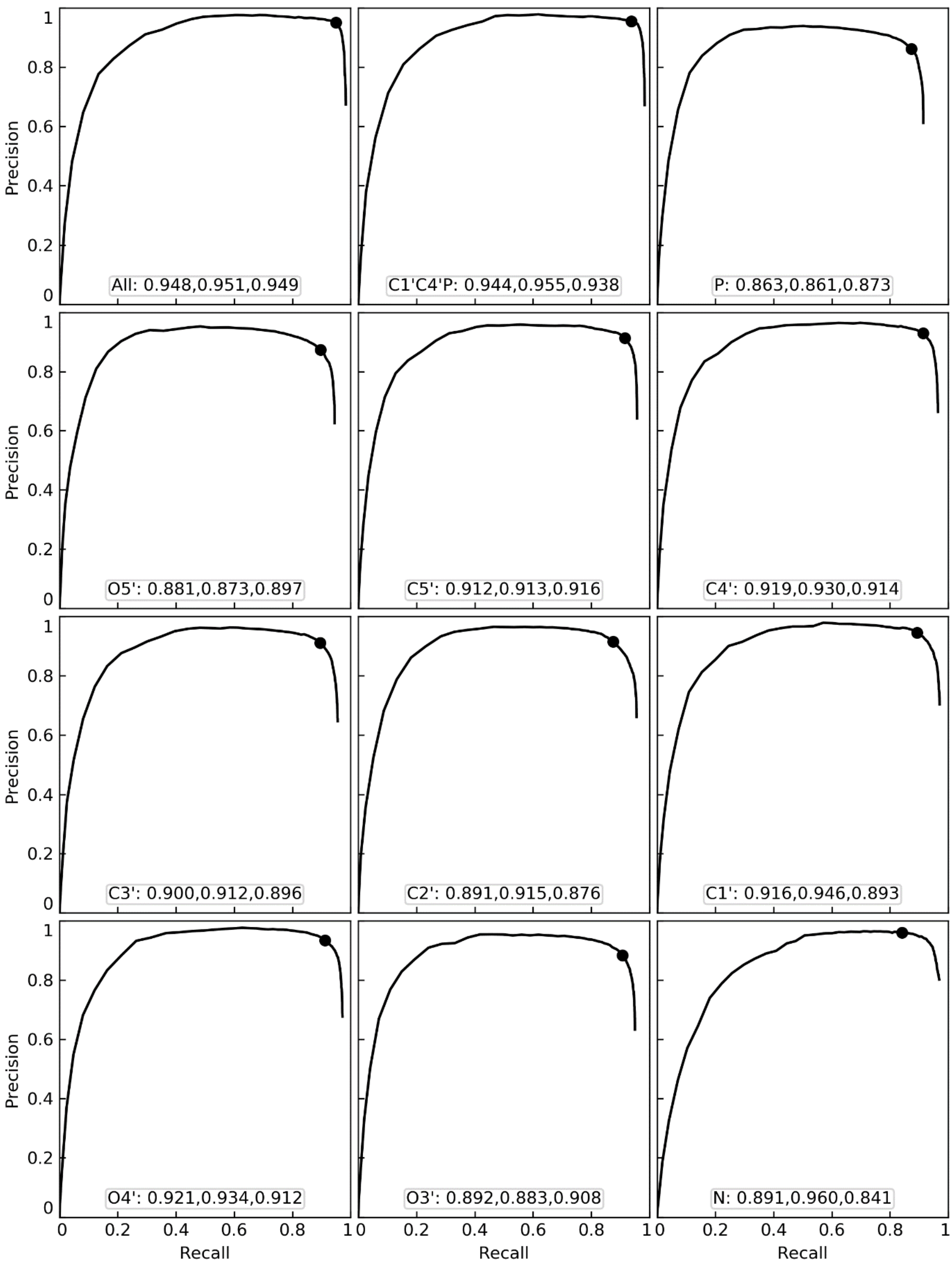


**Figure S2** Precision-recall curves for CSSR assignment for 361 benchmark RNAs. Different panels show different atom types used for assignment. The boxes at lower part of each plot shows the atom type, and the F1-score, precision and recall at CSSR score ≥0.5 (black dots).

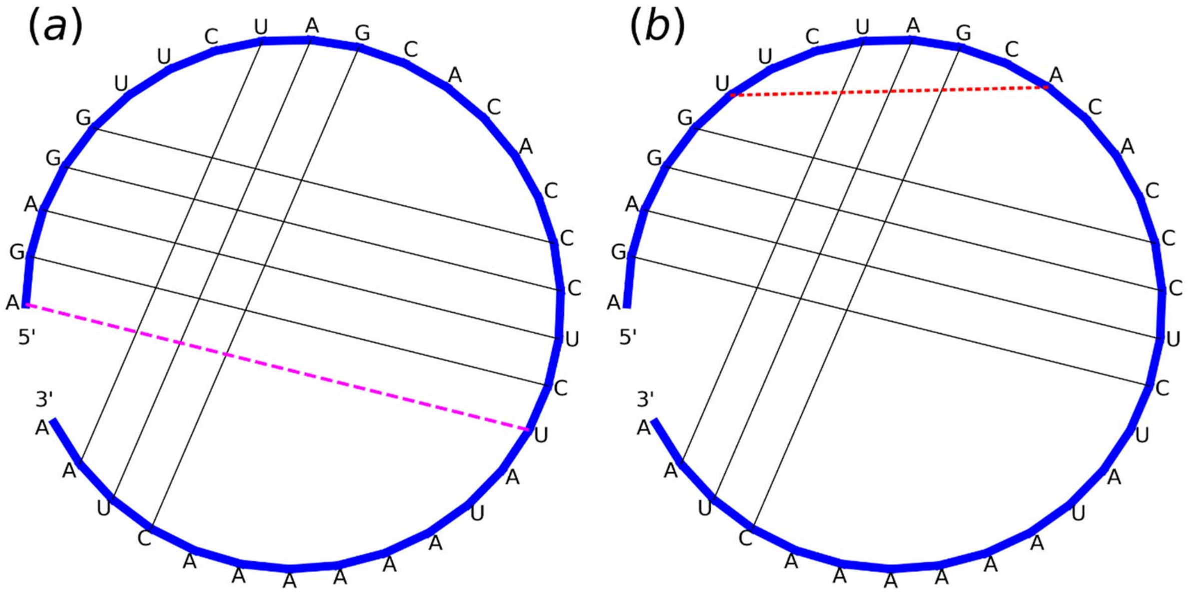


**Figure S3** Assignment of rSS with pseudoknots for PDB ID 3fu2 chain B. (***a***) DSSR assignment. (***b***) CSSR assignment using P atoms only (F1-score=0.875 to DSSR). Base pairs in DSSR but not in final CSSR assignment are shown by magenta dash lines in (***a***). Base pairs in CSSR but not in DSSR are shown by a red dotted line in (***b***). Base pairs common between CSSR and DSSR are shown by black solid lines.
